# Copper and Zinc Homeostasis: Lessons from *Drosophila melanogaster*

**DOI:** 10.3389/fgene.2017.00223

**Published:** 2017-12-21

**Authors:** Juan A. Navarro, Stephan Schneuwly

**Affiliations:** Department of Developmental Biology, Institute of Zoology, University of Regensburg, Regensburg, Germany

**Keywords:** *Drosophila melanogaster*, metal homeostasis, dMTF-1, methallothioneins, copper, zinc, *in vivo* modeling, human diseases

## Abstract

Maintenance of metal homeostasis is crucial for many different enzymatic activities and in turn for cell function and survival. In addition, cells display detoxification and protective mechanisms against toxic accumulation of metals. Perturbation of any of these processes normally leads to cellular dysfunction and finally to cell death. In the last years, loss of metal regulation has been described as a common pathological feature in many human neurodegenerative diseases. However, in most cases, it is still a matter of debate whether such dyshomeostasis is a primary or a secondary downstream defect. In this review, we will summarize and critically evaluate the contribution of *Drosophila* to model human diseases that involve altered metabolism of metals or in which metal dyshomeostasis influence their pathobiology. As a prerequisite to use *Drosophila* as a model, we will recapitulate and describe the main features of core genes involved in copper and zinc metabolism that are conserved between mammals and flies. *Drosophila* presents some unique strengths to be at the forefront of neurobiological studies. The number of genetic tools, the possibility to easily test genetic interactions *in vivo* and the feasibility to perform unbiased genetic and pharmacological screens are some of the most prominent advantages of the fruitfly. In this work, we will pay special attention to the most important results reported in fly models to unveil the role of copper and zinc in cellular degeneration and their influence in the development and progression of human neurodegenerative pathologies such as Parkinson's disease, Alzheimer's disease, Huntington's disease, Friedreich's Ataxia or Menkes, and Wilson's diseases. Finally, we show how these studies performed in the fly have allowed to give further insight into the influence of copper and zinc in the molecular and cellular causes and consequences underlying these diseases as well as the discovery of new therapeutic strategies, which had not yet been described in other model systems.

## Introduction

### *Drosophila melanogaster* as a model organism

The discovery of the first mutant allele of the gene *white* by TH Morgan (Morgan, 1910) meant the beginning of the Age of *Drosophila* as a pivotal model for the study of genetics, developmental biology and, more recently, of neurobiology and human diseases. *Drosophila melanogaster* is a small low-cost organism with a fast life cycle and a relatively short life span (Helfand and Rogina, 2003). Several ground breaking discoveries with strong impact on vertebrate neuroscience have endorsed *Drosophila* during the last century to become the model system of choice for many neuroscientists. Even before the genomic era, the fruit fly was pioneer in unveiling core elements of the nervous system development such as, among many others, Notch, Hedgehog, and Decapentaplegic (Jürgens et al., 1984; Wieschaus et al., 1984). The introduction of the P-element-mediated germline transformation (Rubin and Spradling, 1982) was another milestone in the history of *Drosophila* because it opened a new world of possibilities for genetic manipulations that culminated in 1991 and 1993 with the development of the flippase (FLP) and flippase recognition target (FRT) recombination (Golic, 1991) and the UAS/GAL4 (Brand and Perrimon, 1993) systems. Then, the publication of the first annotated version of *Drosophila* genomic sequence (Myers et al., 2000) confirmed that many human genes involved in all kinds of human diseases had an ortholog counterpart in the fly (Reiter et al., 2001). Moreover, genetic manipulation of those genes has successfully led to molecular, biochemical, tissue and behavioral defects that mirror the human conditions (Botella et al., 2009; Bouleau and Tricoire, 2015; Casci and Pandey, 2015; Xu et al., 2015). A remarkable example is the discovery of the molecular basis of behavior by characterization of genes controlling circadian rhythm and the biological clock (Konopka and Benzer, 1971). The impact of the characterization in the fly of the first clock gene, *period* (Bargiello and Young, 1984; Reddy et al., 1984) has been recently awarded with the Nobel Prize in Physiology or Medicine to Jeffrey C. Hall, Michael Rosbash and Michael W. Young. In addition, *Drosophila* has been instrumental in the characterization of the pathway controlling mitochondrial quality and integrity driven by the Parkinson's disease associated genes Pink and Parkin (Greene et al., 2003; Clark et al., 2006) or providing compelling information even if there was no identified paralogous gene as it is the case for α-synuclein (Feany and Bender, 2000).

The last decades of fly research have boosted the generation of tools in the form of mutants, variants of the UAS-GAL4 system such as the GeneSwitch and the GAL80 inhibitor (Duffy, 2002), the generation of RNAi constructs to target genes in a tissue/cell-specific manner (Dietzl et al., 2007; Ni et al., 2009), the alternative Q system (Riabinina et al., 2015), and, of course, the possibility to create mutants at will by means of the clustered regularly interspaced short palindromic repeat/ endonuclease CRISPR-associated 9 (CRISPR/Cas9) system (Bassett and Liu, 2014). The implementation of these methodologies will allow the fly to continue being the leading model in the analysis of genes involved in human diseases.

### *Drosophila melanogaster* as a model to study metal regulation and homeostasis

The number of manuscripts published using the fly to study the biology of metals is modest. However, the comprehensive work performed and being carried out in the labs of Walter Schaffner, Fanis Missirlis, James Camakaris, Bing Zhou, and Richard Burke has set up the foundations of the field, has provided invaluable translational evidences and has inspired many other fly researchers. *Drosophila* is extremely useful to analyze the regulation and toxicity of iron. Iron is essential to sustain life and it is the most important transition metal due to its cellular roles as electron donor in the oxidative phosphorylation and during photosynthesis. These roles have been detailed during the last decades as well as the impact of iron into several neurodegenerative diseases such as Friedreich's ataxia (FRDA), Parkinson's disease (PD) and neurodegeneration with brain iron accumulation disorders (Poujois et al., 2016). Importantly, the network of genes involved in iron biology and regulation is conserved between vertebrates and *Drosophila* with the exception of the Transferrin Receptor (reviewed in Mandilaras et al., 2013; Calap-Quintana et al., 2017). Fly studies have positively shown that targeting iron metabolism is sufficient to improve diseases conditions in models of FRDA (Navarro et al., 2015; Chen et al., 2016; Soriano et al., 2016), PD (Bonilla-Ramirez et al., 2011; Esposito et al., 2013; Zhu et al., 2016), or Alzheimer's disease (AD) (Rival et al., 2009; Liu et al., 2011; Ott et al., 2015). Besides iron, other functional studies have analyzed the regulation and homeostasis of other heavy metals (Maroni et al., 1986; Al-Momani and Massadeh, 2005; He et al., 2009; Ortiz et al., 2009; Meyer et al., 2014; Rovenko et al., 2014; Ternes et al., 2014; Chandra et al., 2015; Guan et al., 2015; Niehoff et al., 2015). Of special interest is the recent work of Anholt's lab (Zhou et al., 2017) analyzing the genetic networks involved in the resistance to Cadmium and Lead toxicity. This work highlights that, metal homeostasis is much more complex than anticipated and that, 60% of the identified genes have human orthologs. In line with this complexity, fly models have proven that dysfunction of one single metal might be accompanied with dyshomeostasis of other ones. For example, increased aluminum in the fly food also alters iron toxicity (Wu et al., 2012).

In this review, we will focus on the biology of Zinc (Zn) and Copper (Cu). We will summarize and discuss the main findings described in *Drosophila* regarding the cellular and organismal regulation of Zn and Cu with special insight into the nervous system, when possible. Moreover, we will also present a detailed review of the experiments performed with the fly to show the intimate relationship of both metals with human neurodegenerative diseases.

## General facts about Zn and Cu

Although the cellular roles of Cu and Zn are as important as those from iron, several aspects of their metabolism in the fruit fly are not as well characterized. Cu is an important trace element necessary for living organisms because of its redox potential. Free Cu is able to inactivate cellular proteins by directly binding to cysteine residues (Letelier et al., 2009) and can promote oxidative stress and Reactive Oxygen Species (ROS), which will affect other cellular functions (Rotilio et al., 2000). Furthermore, Cu is an essential component of several enzymes (cuproenzymes) involved in diverse cellular processes. The existing literature has highlighted some of them: (i) Mitochondrial Cytochrome C oxidase (COX), a crucial gene for mitochondrial function; (ii) Peptidylglycine α-amidating monooxygenase (PAM) and Dopamine beta (β)-hydroxylase (DBH), involved in the biosynthesis of neurotransmitters; (iii) Lysyl oxidase (LOX), participating in the development of connectivity tissue; (iv) Tyrosinase (TYR), the rate-limiting enzyme in the biosynthesis of melanin, and (v) Superoxide Dismutase I (SOD1), one of the key cellular ROS scavengers (Zlatic et al., 2015; M Fetherolf et al., 2017). Altogether, Cu homeostasis is critical in skin pigmentation, integrity of blood vessels, myelination, maintenance of Purkinje cells, protection against oxidative stress and overall brain function via neurotransmitters (Harris, 2001; Opazo et al., 2014; Zlatic et al., 2015). Interestingly, Cu has also been described as a novel intracellular modulator of signal transduction pathways (Grubman and White, 2014). Accordingly, all this indicates the need of a strict regulation of Cu homeostasis.

Zn is also an essential trace metal with a huge panoply of biological roles and in turn with a strong impact on life. Opposite to Cu and iron, Zn is a redox-neutral element, which might exert antioxidant properties. It is a pivotal element for hundreds of enzymes in three different ways: as a key player of the catalysis (catalytic), as an enhancer of the reaction (coactivator) or by stabilizing the protein (structural) (Vallee and Falchuk, 1993). Zn plays key roles in metabolism of CO_2_ and alcohol, immunological capacity, endocrine response, development of organs and tissues, DNA and protein synthesis and cell division. Zn also participates in the autophagy process (Liuzzi et al., 2014) and therefore, in proteostasis, one of the most important hallmarks of aging. Moreover, Zn has been also described as a signaling molecule itself with important functions in the Bone Morphogenetic Protein (BMP) or the Transforming Growth factor beta (TGF-β) pathways (Osredkar, 2011; Choi and Bird, 2014). Finally, its role as a cofactor for hundreds of transcription factors that contain Zn finger domains is of paramount importance. Remarkably, both, its scarcity and accumulation, lead to cellular damage that might culminate in cell death. Therefore, Zn levels need to be tightly regulated to keep a precise balance and ensure bioavailability of Zn in each cell type.

## MTF-1 and metallothioneins: the first line of defense

Cu and Zn homeostasis relies on its correct acquisition, coordination between import and export, distribution and usage. Understanding the mechanisms underpinning metal sensing and regulation as well as deciphering the regulatory machinery is crucial to fully elucidate the biology of these metals. The transcription factor MTF-1 and the metal-binding proteins named Methallothioneins (Mtns) are common genes involved in the metabolism of Cu and Zn (Choi and Bird, 2014; Krezel and Maret, 2017).

### MTF-1

In mammals, the metal-responsive transcription factor-1 (MTF-1) is the major mediator of protection against accumulation of Zn, Cu and other heavy metals, by inducing the expression of genes that harbor several copies of the metal responsive element (MRE) in their promoters (Stuart et al., 1985). MTF-1 contains six zinc-finger domains and thus, requires Zn for its transcriptional activity. High Zn completely occupies all zinc fingers and then promotes the nuclear translocation of MTF-1. This property suggests, that MTF-1 might be working as a sensing molecule for Zn. Remarkably, MTF-1 also controls the response against oxidative stress, hypoxia, heat shock and even some nutritional alterations. Activation of MTF-1 by these other stressors is not completely elucidated, but seems to be indirect either by a release of Zn from Mtns or by phosphorylation of certain residues (Günther et al., 2012a). Once MTF-1 is in the nucleus, it interacts with other cofactors or additional Zn-dependent transcription factors to establish the specific expression profile according to the stressor (Krezel and Maret, 2017). In mammals, MTF-1 promotes the expression of several Cu and Zn-related genes. Interestingly, it also increases the expression of proteins involved in iron metabolism (ferroportin and hepcidin) or in sensing the cellular redox status (glutamate cysteine-ligase, thioredoxin reductases, and selenoproteins) (Günther et al., 2012a). In mouse, MTF-1 has an essential function since constitutive MTF-1 knockout mice die at day 14 of gestation (Günes et al., 1998), whereas excision of MTF-1 after birth only increased mice susceptibility toward heavy metal stress (Wang et al., 2004). The knockout lethality has been related to a liver dysfunction due to abnormal hepatocyte differentiation rather than to metal toxicity, since knockout of Mtns yielded viable mice (Masters et al., 1994).

**Figure 1:**
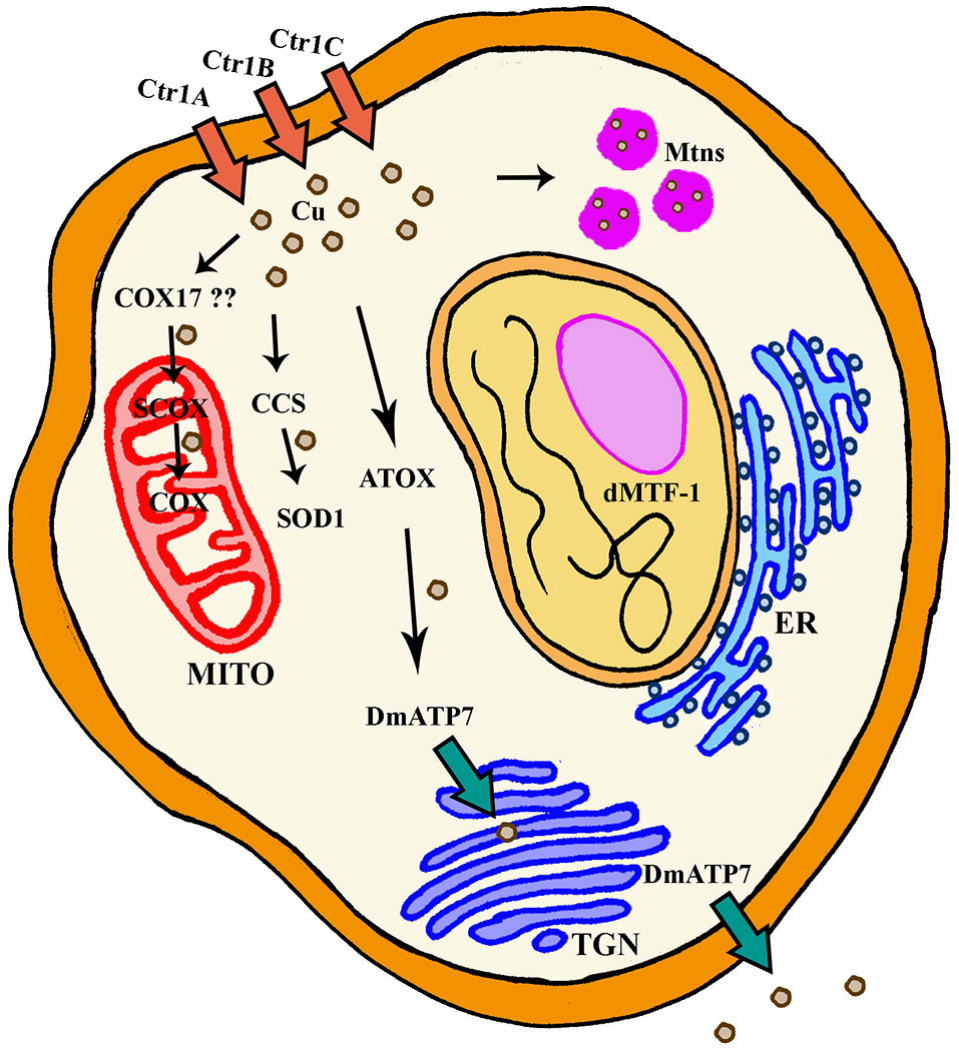
General scheme of cellular Cu homeostasis in *Drosophila melanogaster*. ER, Endoplasmic Reticulum; Mito, Mitochondria; TGN, TransGolgi network.

**Figure 2:**
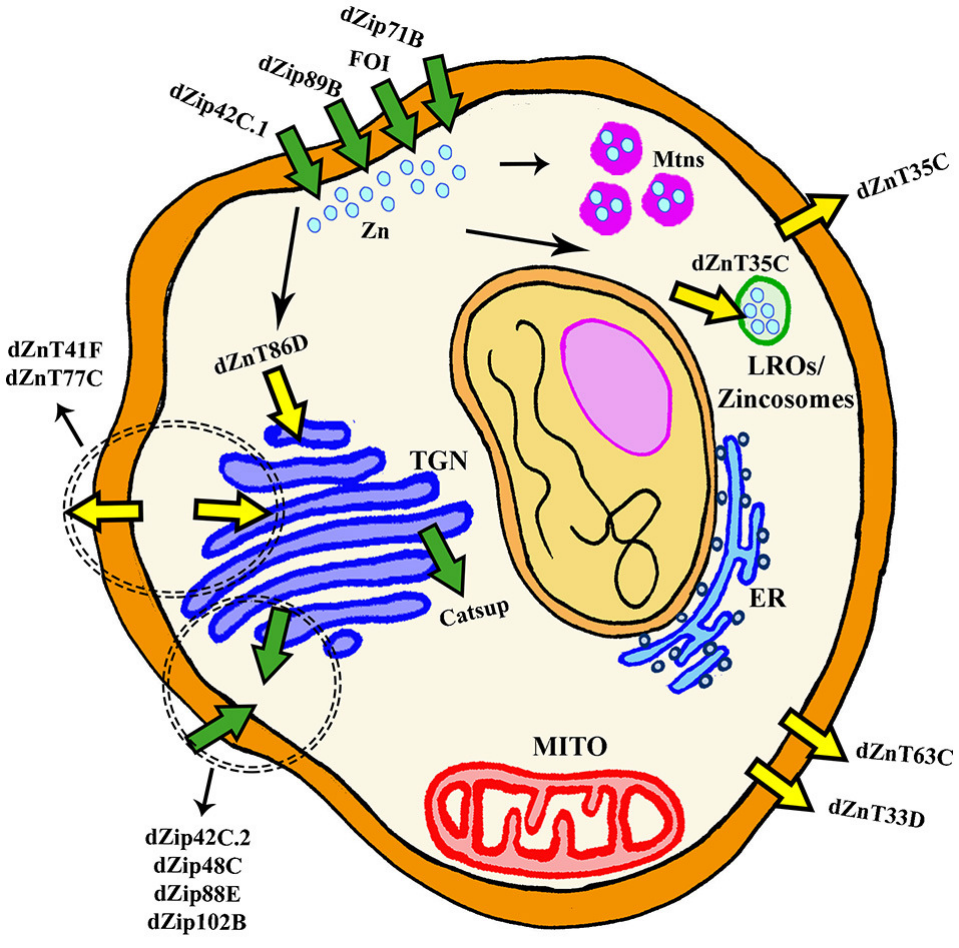
General scheme of cellular Zn homeostasis in *Drosophila melanogaster*. ER, Endoplasmic Reticulum; Mito, Mitochondria; LROs, Lysosomal Related Organelles; TGN, TransGolgi network.

In *Drosophila* (Table 1; Figures 1, 2) such function falls on the shoulders of CG3743 (*dMTF-1*), that was first characterized by Walter Schaffner's lab (Zhang et al., 2001). Surprisingly, although MTF-1 is conserved throughout evolution, the degree of conservation between human and fly genes is only significant in the region containing the six zinc finger domains (81%), whereas the rest of the proteins share only a small 23% of homology (Zhang et al., 2001). Despite these differences, fly dMTF-1 is able to replace the human counterpart in mammalian cell cultures and the human gene restores the tolerance to metals in *dMTF-1* fly null mutants (Balamurugan et al., 2004). The seminal work from Walter Schaffner's group already showed in cell culture, that dMTF-1 was necessary and sufficient to activate the transcription of Mtns via MRE sequences (Zhang et al., 2001). Further works have shown that besides Mtns, iron (ferritin), Cu (*DmATP7* and *Ctr1B*), and Zn (ZnT35C) related genes are also transcriptionally activated by dMTF-1 (Southon et al., 2004; Selvaraj et al., 2005; Yepiskoposyan et al., 2006), suggesting that dMTF-1 participates in the acquisition and storage of metals. Similarly to the human protein, dMTF-1 has additional partners (Günther et al., 2012a) and phosphorylation sites (Gunther et al., 2012b) to allow the discrimination of target genes. Although MTF-1 and dMTF-1 cross-complement, some important differences have been observed. For example, dMTF-1 seems to be nuclear even under non-stress conditions (Gunther et al., 2012b) and it has a C-terminal domain that inhibits its hyperactivation (Gunther et al., 2012b). Moreover, dMTF-1 is essential to regulate Cu homeostasis via a cysteine cluster that works as a copper sensor (Chen et al., 2008).

**Table 1:** *Drosophila* proteins involved in Cu homeostasis (names according to Flybase, http://flybase.org).

**Fly gene**	**Cellular role**	**Human ortholog**	**Related disease fly model**
Metal Transcription factor-1^*^ dMTF-1 (CG3743)	Control of metal-dependent transcription of Mtns and some Cu, Zn and iron genes	*Metal Transcription factor-1 (MTF-1)*	AD (Hua et al., 2011b) MD (Bahadorani et al., 2010a) FRDA (Soriano et al., 2016)
Methallotioneins^*^ MtnA (CG9470) *MtnB (CG4312)* *MtnC (CG5097)* *MtnD (CG33192)* *MtnE (CG42872)*	Cellular metal (mainly Cu) detoxification	*MT-I* *MT-II* *MT-III* *MT-IV*	FRDA (Soriano et al., 2016) AD (Hua et al., 2011b)
*Copper Transporter* *Ctr1A (CG3977)* *Ctr1B (CG7459)* *Ctr1C (CG15551)*	Cellular Cu uptake	*Solute carrier family 31(SCL31A1) hCTR1*	AD (Lang et al., 2013) HD (Xiao et al., 2013) MD (Bahadorani et al., 2010a) PD (Saini et al., 2010)
*ATPase 7* *DmATP7 (CG1886)*	Cellular Cu export Cu delivery	*ATPase copper transporting α and β* (*ATP7A and ATP7B*)	AD (Lang et al., 2013) HD (Xiao et al., 2013) MD (Bahadorani et al., 2010a; Southon et al., 2010; Mercer et al., 2017) WD (Mercer et al., 2017)
*Antioxidant 1 copper chaperone* *Atox1(CG32446)*	Chaperone. Cu delivery to DmATP7	*Antioxidant 1 copper chaperone (ATOX1)*	AD (Sanokawa-Akakura et al., 2010) FRDA (Soriano et al., 2016)
*Copper Chaperone for superoxide dismutase* *Ccs (CG17753)*	Chaperone. Cu delivery to SOD1	*Copper Chaperone for superoxide dismutase (CCS)*	Not tested
*Synthesis of cytochrome c oxidase* *Scox (CG8885)*	Chaperone. Cu delivery to cytochrome c oxidase	*Cytochrome c oxidase copper chaperone (SCO1)*	Not tested

*Gene symbols for human genes follow the regulation of Human Genome Organization Gene Nomenclature Committee (http://www.genenames.org)*.

* *dMTF-1 and Mtns have been added in this table since their main role in Drosophila melanogaster is the detoxification of Cu accumulation. AD, Alzheimer's disease; FRDA, Friedreich's ataxia; HD, Huntington's disease; MD, Menkes disease; PD, Parkinson's disease; WD, Wilson's disease*.

Following the classical reverse genetic approach, mutant flies and flies overexpressing *dMTF-1* have been generated to analyze its functions in *Drosophila*. Opposite to mouse, mutant *dMTF-1* flies did not show any developmental defect, but presented a shorter longevity (Bahadorani et al., 2010b). Remarkably, they display an increased sensitivity toward accumulation of metals due to the lack of induction of the protective Mtns and the consequent accumulation of free metals in toxic concentrations (Egli et al., 2003, 2006b; Bahadorani et al., 2010b). In this line of events, *dMTF-1* overexpression in central and peripheral nervous systems extended longevity under normal conditions, although no effect was observed upon expression in the gut (Bahadorani et al., 2010b). These results indicate that the control of metal homeostasis has a crucial tissue-specific component.

Cu and Zn show an unusual relationship with *dMTF-1*. On the one hand, *dMTF-1* mutants are more sensitive to both Cu scarcity and supplementation, whereas overexpression increases resistance in both situations (Selvaraj et al., 2005; Bahadorani et al., 2010b). This dual role in detoxification and acquisition is likely related to the simultaneous control of the expression of the Cu importer *Ctr1B* and the Cu exporter *DmATP7* by dMTF-1 (Selvaraj et al., 2005; Yepiskoposyan et al., 2006; Burke et al., 2008; Bahadorani et al., 2010b). Activation of both might affect metal distribution and balance in a tissue-specific manner. On the other hand, dMTF-1 adult mutant flies are not sensitive to toxic concentrations of Zn. However, overexpression of dMTF-1 ubiquitously, in the gut or in the neurons makes the flies hypersensitive to Zn. Again, the explanation may rely in the induction of, at least, the zinc exporter ZnT35C by dMTF-1 (Yepiskoposyan et al., 2006). This induction will trigger aberrant ion distribution with depletion in some tissues and accumulation in others. In agreement, the overall amount of Zn was reduced in flies overexpressing dMTF-1 (Bahadorani et al., 2010b). These results suggested that nervous system and gut were particularly sensitive to perturbation of Zn homeostasis (Bahadorani et al., 2010b).

### Metallothioneins

Mtns are ubiquitous, small cystein-rich proteins present from yeast to human with the ability to bind heavy metal ions and with thiol reactivity. Mtns are able to modulate their redox state in order to regulate metal bioavailability and buffer deleterious accumulation. Mammalian Mtns also respond to additional factors such as hormones, cytokines or oxidative stress. In humans up to 12 functional Mtns have been characterized, being MT-I to IV the most important family members (Palmiter, 1998; Krezel and Maret, 2017), whereas only five (A–E) have been found in the fruit fly (Egli et al., 2006a; Atanesyan et al., 2011). They carry out an essential metal detoxification because mutations and polymorphisms in Mtns are associated to human diseases ranging from diabetes, cancer, autoimmune diseases, neurodegenerative disorders and autism (Laukens et al., 2009; Raudenska et al., 2014). In mice, mutations in two Mtns do not induce lethality, but confer sensitivity toward Zn (Kelly et al., 1996).

Already in 1986, experiments in *Drosophila* larvae suggested the existence of genes with metal binding properties, whose expression was inducible by increasing concentration of metals (Maroni et al., 1986). Later on, the spectrum of stressors able to activate the expression of Mtns in flies was extended to thermal and oxidative stress (Bonneton et al., 1996). MtnA and B (firstly named as Mtn and Mto, respectively) were the first ones identified (Durliat et al., 1995) followed by MtnC and D (Egli et al., 2003) and more recently a fifth member (MtnE) was added (Atanesyan et al., 2011). In flies, all five Mtns (Table 1, Figures 1, 2) depend on dMTF-1 (Egli et al., 2003; Southon et al., 2004; Atanesyan et al., 2011) and are broadly conserved among the *Sophophora* and *Drosophila* subgenus (Guirola et al., 2011). MtnA shows the highest expression in all developmental stages (Atanesyan et al., 2011; Graveley et al., 2011). In larvae, all Mtns are expressed in larval gut, fat body, Malpighian tubules or salivary glands (Durliat et al., 1995; Egli et al., 2006b; Atanesyan et al., 2011). Increasing metal content in the fly food enhanced the levels of Mtn in those tissues and expanded the pattern to new regions. Expression in adult tissues is very poorly described with the exception of the work from Durliat and collaborators (Durliat et al., 1995). In this case, it was shown that MtnA and B were expressed in the adult cardia, gut or Malpighian and no expression was detected in other tissues. However, and in light of the increasing importance of metal detoxification in human diseases, a deeper analysis of Mtn expression in adults using new technical approaches and the fly tool box is of high interest. In this sense, we have detected robust expression of most of the Mtns in adult heads by Real Time-PCR (our unpublished observations).

Metal specificity is a critical issue regarding the biology of Mtns. Single, double, triple and quadruple mutants of MtnA-D (qMtn) have been generated to address this question (Egli et al., 2006a,b). Similarly to MTF-1, all Mtn mutants are viable and fertile. The analysis of developmental hypersensitivity to toxic metal concentrations in the different mutant combinations has provided valuable information. Those experiments revealed that MtnA and B are, respectively, essential to detoxify Cu and Cd. MtnC and D did not seem to play a major role in protection (in agreement with their developmental expression) and Zn only had a minor impact even in the qMtn background. In contrast to *dMTF-1* mutants, qMtn flies were not sensitive to Cu depletion, suggesting that Mtns are not essential for Cu import or to transfer Cu. Regarding adults, qMtn flies show a strong hypersensitivity toward Cu, but no information about other metals has been reported. MtnE responds to a broader range of metal including Cu, Zn, cadmium, silver and mercury (Pérez-Rafael et al., 2012). In accordance to this less specific induction, MtnE displays a broader expression pattern. This result might indicate that it could be important in a context, in which the other Mtns fail. This hypothesis is supported by the induction of MtnE expression in qMtn mutant (Atanesyan et al., 2011). Interestingly, a constant exposure to metals attenuates the initial boost of Mtn expression and this effect has not been detected in other dMTF-1 target genes (Egli et al., 2006b). The binding abilities of all five Mtns are different and nicely correlate with their responsiveness to toxic metal concentrations. All show low affinity to Zn and higher affinity to bind Cu and cadmium (Egli et al., 2006a; Pérez-Rafael et al., 2012). Anyways, quantification of expression levels of MtnB and C either by Real-Time PCR or by induction of a genetic reporter has been successfully used to monitor Zn levels in flies (Georgiev et al., 2010; Saini and Schaffner, 2010; Qin et al., 2013; Yin et al., 2017). We can conclude that fly Mtns act primarily as Cu-thioneins in comparison to mammalian counterparts (Zn-thioneins) although several aspects of Mtn's cellular functions still need to be fully elucidated.

## Regulation of Cu metabolism

Several proteins have already been described to participate in the Cu metabolism. In short, Cu is mainly imported via the copper transporter CTR1. Cu is then distributed by the copper chaperones, small proteins that deliver the metal to cuproenzymes such as SOD1 and cytochrome c oxidase, or to other members of the Cu regulatory network named ATP7A and ATP7B. These two P-type ATPases are also Cu donors to other Cu-containing enzymes and participate in Cu extracellular delivery (Madsen and Gitlin, 2007). *Drosophila* has been used to study Cu biology since the early fifties (Poulson et al., 1952). *Drosophila* S2 cells express all classical genes involved in regulation (import, delivery and export) of Cu and their silencing altered Cu homeostasis as predicted, suggesting a conserved role. For example, knockdown (KD) of *Ctr1B* reduced Cu uptake and content, whereas downregulation of *DmATP7* induced Cu accumulation due to a deficient efflux. As a result, the systemic Cu homeostasis is very similar between humans and *Drosophila melanogaster* (Southon et al., 2013a). Figure 1 and Table 1 summarize the key fly proteins involved in Cu metabolism. One of the main handicaps of the studies about Cu biology is the lack of proper and direct ways to measure Cu and to monitor differences either in the amount or in the distribution of the metal. Richard Burke's lab has very elegantly and nicely showed that synchrotron x-ray fluorescence microscopy is able to detect Cu alterations in a small organism such as *Drosophila* even in some tissues including the fly brain (Lye et al., 2011).

### Cu uptake

Copper transporter 1 (CTR1) is the main responsible of cellular import of Cu. CTR1 belongs to the SLC31 family of transporters and forms a trimeric channel to allow Cu uptake (Nevitt et al., 2012). The fly genome contains three different orthologs (Figure 1): *Ctr1A, Ctr1B, Ctr1C* (Zhou et al., 2003). *Ctr1A* is highly expressed throughout development, whereas *Ctr1B* seems to be only relevant in late embryo and larvae with very strong expression in enterocytes (in order to uptake Cu from food). *Ctr1C* is very lowly expressed in all developmental stages and the expression seems to be mainly focused in male gonads (Zhou et al., 2003; Hua et al., 2010). All three proteins seem to be mainly localized in the plasma membrane (Selvaraj et al., 2005; Turski and Thiele, 2007; Binks et al., 2010; Steiger et al., 2010). In agreement, they are able to stimulate Cu import into *Drosophila* S2 cells (Zhou et al., 2003) and also *in vivo* (Balamurugan et al., 2007; Hua et al., 2010). Ubiquitous or panneuronal overexpression of any of the three genes is developmentally lethal, whereas eye-specific overexpression triggered a rough eye phenotype accompanied by neuronal degeneration (Balamurugan et al., 2007; Hua et al., 2010; Lang et al., 2013). This retinal degeneration is clearly related to Cu availability, since reducing Cu content in retinal neurons by increasing efflux (Balamurugan et al., 2007), enhancing Cu chelation by Mtns (Egli et al., 2006a), or with bathocuproine disulfonate (BCS) (Balamurugan et al., 2007; Hua et al., 2010) can rescue the phenotype.

*Ctr1A* seems to be the most important of all fly copper importers, since it displays the broadest expression pattern and its complete loss-of-function triggers developmental arrest (Turski and Thiele, 2007; Hua et al., 2010). In agreement with a reduced Cu import, *Ctr1A* deficient larvae and adults show lower activity of Cu-requiring enzymes such as Cytochrome c Oxidases (*Coxs*), tyrosinase (*Tyr*) and peptidylglycine-hydroxylating monooxygenase (*Phm*). This resulted in lower pigmentation of mouth hooks and body, and reduced amidation of neurotransmitters (Turski and Thiele, 2007; Binks et al., 2010). Importantly, Cu-supplementation in the food (Turski and Thiele, 2007; Hua et al., 2010) or genetic manipulation of intracellular copper levels (Binks et al., 2010) improved some of the defects. Furthermore, the human counterpart hCtr1 also improved loss of *Ctr1A* conditions, suggesting a strong functional equivalence between both proteins (Hua et al., 2010). Due to the lethality associated to the complete depletion of *Ctr1A*, tissue-specific experiments have also been performed. KD in the dorsal vessel generated a severe cardiomyopathy in the flies (Kim et al., 2010). This result demonstrates that cardiac defects present in several models of systemic Cu deficiency are a result of a tissue-specific effect. It is likely that, in a tissue highly dependent on mitochondria, strong reduction of *Cox* activity is underlying the cardiac defect. *Ctr1A* depletion in the nervous system showed that it is required for neuronal development (Binks et al., 2010). Interestingly, KD of *Ctr1A* in the protothoracic gland induced phenotypes reminiscent to a reduced Ras signaling. Accordingly, downregulation of *Ctr1A* was able to rescue the overactivation of Ras (Turski et al., 2012). *Ctr1B* is the only Cu importer identified as a direct target of dMTF-1 (Southon et al., 2004; Selvaraj et al., 2005). *Ctr1B* is upregulated by Cu deprivation (Zhou et al., 2003) in order to maintain the correct systemic Cu amount and, unexpectedly, also by toxic accumulation of mercury and cadmium (Balamurugan et al., 2009). Mutants are viable but show hypopigmentation due to defects in the activity of the Cu-containing enzyme *Tyr*. Since those mutants do not accumulate Cu, they fail to induce the expression of Mtns (Zhou et al., 2003; Balamurugan et al., 2007). This defect is really Cu specific since mutants successfully promoted Mtns expression upon feeding with cadmium (Balamurugan et al., 2007). However, under conditions of Cu scarcity, mutants are unable to complete development (Zhou et al., 2003). In agreement with the specific expression pattern in the fly gut, a panneuronal KD of *Ctr1B* does not affect cellular integrity (Binks et al., 2010). *Ctr1C* is the least studied and its expression seems to be restricted to the maturing spermatocytes and the mature sperm. *Ctr1C* mutants are viable but their fertility is severely compromised in a Cu-dependent manner (Steiger et al., 2010). *Malvolio* (*Mvl*), the *Drosophila* homolog of *Divalent metal transporter-1* (*DMT1*) is mostly involved in the transport of dietary iron in the gut (Folwell et al., 2006) but also seems to influence Cu content in *Drosophila* S2 cells (increased when overexpressed and reduced when depleted) and in the fly gut (Southon et al., 2008). Mutant individuals are viable but sensitive to increased levels of Cu in the food. Since its function might be redundant with members of the Ctr1 family, it is logical that the loss-of-function does not cause a systemic Cu deficiency.

### Cu efflux

In mammals, the responsibility to secrete Cu from cells relies on two P-Type ATPases, ATP7A, and ATP7B. They both belong to a group of genes that hydrolyze ATP to pump substrates across membranes (Zlatic et al., 2015). The fly genome only encodes one ortholog of mammalian ATP7A and B, DmATP7 (Figure 1) which displays a high homology (>45%) to both ATPases (Southon et al., 2004). However, comparative genomic analysis of 12 *Drosophila* species shows that DmATP7 contains all the motifs existing in ATP7A required for localization and retention in the basolateral membrane, whereas ATP7B targeting motifs are not present (Southon et al., 2010). ATP7A has been described as a multitasking protein. It seems to be initially located in the transgolgi network (TGN), where it participates in the transfer of Cu into Cu-containing proteins and it is relocated to the plasma membrane upon increasing concentrations of Cu (Kaler, 2011), where it is involved in cellular efflux of Cu. Several functional reporter constructs (*DmATP7-lacZ, EYFP-DmATP7, UAS-DmATP7FLAG, gDmATP7:GFP*) have been used to study the endogenous expression pattern of DmATP7. DmATP7 is highly expressed at the basolateral membrane of the Cu cells of the midgut and in the central, peripheral and enteric nervous system in all developmental stages. It is also expressed in the larval mouthparts, developing tracheae, the gut and in the malpighian tubules but not in the optic lobes, salivary glands and fat body. In the adult nervous system the expression in peptidergic neurons is of special importance due to the role of Cu in the biosynthesis of amidated neurotransmitters (Norgate et al., 2006; Burke et al., 2008; Sellami et al., 2012; Mercer et al., 2017). In all cases, fusion proteins show clear localization at the plasma membrane. Importantly, DmATP7 presents a Cu-inducible and dMTF-1 dependent expression (Burke et al., 2008; Mercer et al., 2017). It is known that the Cu-induced trafficking of mammalian ATP7A and ATP7B from the TGN toward the plasma membrane is critical for their role in Cu homeostasis (Kaler, 2011). Unexpectedly, this trafficking was not observed in cultured embryonic S2 cells, larval neuronal Bm3-c2 cells (Southon et al., 2010) or *in vivo* in larval midgut cells (Burke et al., 2008; Mercer et al., 2017) with any of the DmATP7 constructs. However, DmATP7 is able to translocate from the TGN to the plasma membrane when introduced in mammalian cells (Southon et al., 2010). Therefore, fly models still need to clarify whether such translocation is not required/necessary in flies or whether just current methodological limitations avoid its detection. In agreement with the expected role in Cu transport and export, coexpression of DmATP7 counteracted the phenotypes induced by the overexpression of the Cu importer (*Ctr1B*) in the *Drosophila* retina (Balamurugan et al., 2007). Panneural overexpression of DmATP7 induced an altered morphology of neuromuscular junctions (Comstra et al., 2017) followed by a partial developmental lethality and a high percentage of unexpanded wings in the surviving adults due to a deficient activity of the corresponding neurotransmitters (Hwang et al., 2014). Similarly, ubiquitous overexpression induced body hypopigmentation due to the impairment of the Cu-requiring enzyme *Tyr* (Norgate et al., 2006). Since all these effects are rescued by Cu-supplemented food, we can conclude that overexpression also results in Cu deficiency due to increased efflux (Norgate et al., 2006; Hwang et al., 2014).

### Cu chaperones

In addition to transporters of Cu into and out of the cells, there is a third group of proteins with critical roles in Cu homeostasis. They are the so called “Cu chaperones” since their main role is to deliver Cu into aproproteins of cuproenzymes (Palumaa, 2013). *Drosophila* orthologs (Figure 1) of the mammalian Cu chaperones *ATOX1, CCS, COX17* and *SCO1*, and *SCO2* are found to be expressed in *Drosophila* S2 cells (Southon et al., 2004). *Atox1* is the fly homolog of the mammalian *ATOX1*, which delivers Cu to ATP7A and ATP7B. In agreement, loss of *Atox1* function in the flies phenocopies most of the defects of DmATP7 mutant flies (Hua et al., 2011a). *CCS* is the chaperone that delivers Cu to SOD1 (Cu/Zn superoxide dismutase). *Ccs* mutant flies display low levels of SOD1 activity, but also reduced levels of SOD1 protein. This important evidence suggests that *Ccs* plays a role in the stability of SOD1 (Kirby et al., 2008). SCO proteins are the Cu chaperones in charge of delivering Cu to the mitochondrial cytochrome c oxidase (COX). The *Drosophila* genome only encodes a single ortholog named *Scox* that is expressed during the whole development. Correspondingly, null mutants show a severely reduced COX activity that triggers developmental arrest at L2 stage. Hypomorphic alleles affect locomotion and female fertility, in agreement with its high expression in the egg chambers (Porcelli et al., 2010). It is likely that mitochondrial dysfunction is underpinning the loss-of-*scox* defects. However, additional studies will be needed to find out the complete mitochondrial mechanism. Unfortunately, although homologs of mammalian COX11 and COX17 have been found *in silico*, CG9065 and CG31648, and at least one of them is expressed in S2 cells (Southon et al., 2004), there is no report addressing their role in the Cu homeostasis or in the mitochondrial function.

### Cuproenzymes and other Cu-related genes

The classical enzymes described to require Cu in their activity, which have fly orthologs are: *Lysyl oxidase*, involved in connective tissue (Molnar et al., 2005); *Peptidylglycine-*α*-hydroxylating monooxygenase*, involved in amidation of peptides (Hwang et al., 2014); Multi-copper oxidases MCO1 and MCO3, involved in iron homeostasis (Mandilaras et al., 2013); Tyramine β-Hydroxylase, involved in neurotransmitter synthesis (Monastirioti et al., 1996); tyrosine hydroxylase, involved in synthesis of melanine and dopamine (Wright, op. 1987) and Zn/Cu superoxide dismutase (SOD1), involved in protection against oxidative stress (Phillips et al., 1989). SOD1 is by far the most studied in the fly, due to its strong relation with oxidative stress, aging and amyotrophic lateral sclerosis (ALS) (Renton et al., 2014; Casci and Pandey, 2015).

Moreover, *in vivo* screenings and microarray experiments have helped to identify new candidate genes involved in the regulation of Cu. Syntaxin 5 was identified in a screening of genes that modulate mortality on excess Cu (Norgate et al., 2007). It is a soluble NSF attachment protein receptor (SNARE) gene involved in intracellular vesicle trafficking with high impact on Cu uptake (Norgate et al., 2010). Other similar examples are CG14036, CG11825, CG14545 (Norgate et al., 2007). VhaPPA1-2 and bib are a couple of genes that were first discovered as required for cuticle pigmentation and lately characterized as novel mediators of Cu biology that facilitate the membrane localization of Cu transporters (Mummery-Widmer et al., 2009; Wang et al., 2014). ADP-ribosylation factor 1, Adf1, contributes to maintain the golgi structure (Southon et al., 2011). Glutamatecysteine ligase catalytic subunit gene (Gclc) is the rate-limiting enzyme in glutathione (GSH) formation. GSH is a key cellular antioxidant molecule that also plays a role in Cu regulation (Mercer et al., 2016).

## Regulation of Zn metabolism in *Drosophila*

Although Zn is an essential metal involved in a large panoply of key cellular processes, the *in vivo* physiological roles of Zn transporters are still not well characterized. In humans and in *Drosophila*, Zn homeostasis is mainly mediated by two large families of zinc transporters, the SLC39A or Zip (which import zinc into the cytoplasm) and the SLC30A or ZnT (which remove zinc from of the cytoplasm). Importantly, they are conserved throughout evolution from humans to flies (Lye et al., 2012). *In silico* and *in vivo* studies have identified up to 17 members of both families in the fly (Lye et al., 2013), whereas 24 proteins belong to those groups in mammals (Lichten and Cousins, 2009). In general, all Zn transporters in the fly are named by their cytological location (Yepiskoposyan et al., 2006) with the exception of *catecholamines up* (*catsup*) and *fear of intimacy* (*foi*) that retain their original names.

In the fly, most of the work to characterize the expression and function of dZip and dZnT genes has been performed in the Malpighian tubules and in the midgut because Zn mainly accumulates in the tubules and the gut is the primary site for the absorption of nutrients in *Drosophila* (Schofield et al., 1997). Therefore, global and systemic changes have been investigated in detail, but little is still known about local redistribution and availability of Zn within a given tissue or organ and even within the cell. Approaches based on genetic interactions have played an instrumental role in the identification of new genes involved in Zn homeostasis and in the analysis of the cellular and systemic roles of each transporter. Richard Burke's lab developed a brilliant strategy for this aim. They initially found that simultaneous overexpression of the main importer (*dZip42C.1*) and silencing of the main exporter (*dZnT63C*) triggered Zn entrapment in the cytosol, that lead to a Zn toxic phenotype (named Ztox) characterized by either retinal degeneration and loss of eye pigmentation when carried out in the developing eye (*gmr*-GAL4) or by scutellum loss, thorax cleft, hypopigmentation and bristles misaligned when performed in the developing thorax (*pnr*-GAL4) (Lye et al., 2012). Similarly, overexpression of *dZnT86D* or *dZip71B* also induce changes in the eye morphology and pigmentation (Dechen et al., 2015). In parallel, Bing Zhou's lab conducted similar studies within the gut (Qin et al., 2013; Yin et al., 2017). Importantly, all these phenotypes were modulated (rescued or worsened) by chemical or genetic manipulation of Zn levels (Lye et al., 2012, 2013; Qin et al., 2013; Dechen et al., 2015; Yin et al., 2017). The genetic interactions showed which combinations of Zn transporters modified the eye morphology, the thorax pigmentation or the response toward Zn overload/depletion. All this information along with the subcellular localization described by reporter lines has allowed dividing the Zn transporters into four subgroups.

Table 2 and Figure 2 recapitulate all fly genes involved in Zn transport except dZnT49B. It shows sequence similarity to the *hZIP9* gene but no other information about the gene is available and it cannot be properly classified. It is important to note, that the results from the interactions between the Zn toxic genotypes and the collection of RNAi constructs provides additional evidences about the expression pattern of the Zn transporters and complements the information available in the Flyatlas (flyatlas.org). In this sense, the results already reported (Lye et al., 2012, 2013; Dechen et al., 2015) suggest that almost all transporters are expressed in the developing eye, in the thorax or in the PNS.

**Table 2:** *Drosophila* proteins involved in Cu homeostasis (names according to Flybase, http://flybase.org).

**Fly gene**	**Cellular role**	**Human ortholog**	**Related disease fly model**
*dZip42C.1 (CG9428)* *dZip89B (CG6898)* *dZip71B (CG10006)* *foi (CG6817)*	Cellular Zn uptake	*hZIP1, hZIP2, hZIP3 hZIP1, hZIP2, hZIP3 hZIP5 hZIP6, hZIP10*	AD (Lang et al., 2012; Huang et al., 2014) FRDA (Soriano et al., 2016) KSD (Chi et al., 2015; Yin et al., 2017) PD (Saini and Schaffner, 2010)
*dZip42C.2 (CG9430)* *dZip88E (CG4334)* *Catsup (CG10449)* *dZip48C (CG13189)* *dZip102B (CG2177)*	Cytosolic Zn uptake from extracellular matrix and from organelles	*hZIP1, hZIP2, hZIP3 hZIP1, hZIP2, hZIP3 hZIP7 hZIP11 hZIP9*	FRDA (Soriano et al., 2016) PD (Chaudhuri et al., 2007)
*dZnT63C (CG17723)* *dZnT33D (CG31860)*	Cellular Zn export	*hZnT1, hZnT10 hZnT2, hZnT3, hZnT4, hZnT8*	AD (Huang et al., 2014) FRDA (Soriano et al., 2016) KSD (Chi et al., 2015; Yin et al., 2017) PD (Saini and Schaffner, 2010)
*dZnT35C (CG3994)* *dZnT41F (CG11163)* *dZnT77C (CG5130)* *dZnT86D (CG6672)*	Cytosolic Zn export outside of the cell or into cellular organelles	*hZnT2, hZnT3, hZnT4, hZnT8 hZnT2, hZnT3, hZnT4, hZnT8 hZnT1, hZnT10 hZnT5, hZnT6, hZnT7*	FRDA (Soriano et al., 2016) KSD (Chi et al., 2015; Yin et al., 2017) PD (Saini and Schaffner, 2010)

*Gene symbols for human genes follow the regulation of Human Genome Organization Gene Nomenclature Committee (http://www.genenames.org). AD, Alzheimer's disease; FRDA, Friedreich's ataxia; KSD, Kidney Stones disease; PD, Parkinson's disease*.

### Importers of extracellular Zn

The proteins, which have been classified as pure Zn importers into the cytosol from the extracellular matrix are dZip42C.1 (dZip1), dZip89B, dZip71B and FOI (Figure 2). All genes of this category are localized in the plasma membrane (van Doren, 2003; Dechen et al., 2015). dZip42C.1 has been described as the main protein responsible for dietary Zn absorption in the midgut enterocytes (Lang et al., 2012; Qin et al., 2013). In parallel to this function, dZip42C.1 also participates in Zn import into the adult nervous system (Lang et al., 2012). Unexpectedly, reduction of *dZip42C.1* in the gut did not trigger any deleterious effect, even under Zn depletion conditions. This strongly suggests the presence of other dZip proteins performing Zn uptake in the gut (Qin et al., 2013). Bioinformatic analysis showed that dZip89B and dZip42C.2 (that will be introduced in the next section) were the best candidates. In agreement, dZip89B increases cytosolic Zn levels when overexpressed (Lye et al., 2013; Dechen et al., 2015). Furthermore, flies lacking dZip89B are normal, although they show reduced Zn levels in the gut along with upregulation of dZip42C.1 and dZip42C.2. In addition, dZip89B has a broader expression pattern and works as a low-affinity Zn transporter (Richards et al., 2015). All this might explain the lack of sensitivity of dZip89B mutants to Zn deprivation. dZip71B performs the opposite role to dZip42C.1 and its co-workers (dZip42C.2 and dZip89B). It is mainly expressed in the Malpighian tubules and imports Zn from the body for detoxification and disposal. Therefore, it is instrumental in the excretion of Zn from the organism and accordingly, silencing of *dZip71B* triggers Zn accumulation in the body and these flies are more sensitive to Zn overload (Yin et al., 2017). When overexpressed, *dZip71B* acts as a very efficient and potent Zn transporter able to create a toxic Zn accumulation on its own (Richards and Burke, 2015). Interestingly, both reports highlight cell-specific regulation of Zn metabolism.

FOI is not expressed in the gut, but is one of the cellular Zn importers in several other tissues (probably cooperating with dZip42C.1). The gene *fear of intimacy* (*foi*) showed up in genetic screens aiming to identify mutations affecting the development of fly gonads and trachea (van Doren, 2003; Mathews et al., 2005), embryonic nervous system (Pielage et al., 2004), and fly muscles (Carrasco-Rando et al., 2016). FOI is able to transport Zn in yeast and mammalian cells (Mathews et al., 2005). The Zn-related activity of *foi* is required for the stability of the cell-adhesion protein E-cadherin, that is essential for the proper formation of the gonads (Mathews et al., 2005, 2006). In the myoblast development, the expression of target genes of zinc-dependent transcription factors, such as Minc and Kruppel, was severely altered in *foi* mutants. In agreement, the phenotypes could be rescued by overexpression of other dZip genes with the same cellular localization such as *dZip42C.1* and *dZip71B* (Carrasco-Rando et al., 2016). All these results support the role of *foi* as a Zn transporter and the impact of its loss-of-function in the activity of zinc-finger transcription factors.

### Importers of intracellular-stored Zn

This category includes proteins that, besides having some capacity to uptake Zn from the outside of the cell, they have been shown to remove Zn also from intracellular compartments. This group encompasses the following proteins dZip42C.2 (dZip2), dZip88E, CATSUP, dZip99C, dZip48C, and dZip102B (Figure 2). dZip42C.2 is another Zn importer from the lumen into the enterocytes (Qin et al., 2013). Flies with reduced dZip42C.2 levels were normal but they display severe phenotypes (more than those with reduced dZip42C.1) including developmental arrest under low zinc conditions. This suggests, that dZip42C.2 might play more crucial roles than its counterpart dZip42C.1. Interestingly, expression levels of both seem to be regulated by Zn levels in the enterocytes (Qin et al., 2013). dZip88E was initially thought to be the fourth member of this group formed by dZip42C.1, dZip42C.2, and dZip89B to absorb Zn from diet (Lye et al., 2012). However, its recent characterization (Richards et al., 2017) and the results from genetic interactions (Dechen et al., 2015), suggest a completely different role. It is expressed in a small subset of cells in the gut and larval CNS displaying both, plasma membrane and intracellular localization (Dechen et al., 2015; Richards et al., 2017). Interestingly, even with this restrictive expression, mutant flies are hypersensitive toward Zn. It seems that dZip88E expression in these cells acts as a surveillance system to detect Zn toxicity and elicit a systemic response to counteract it.

*Catsup* is another gene that was isolated long before it was described as a dZip gene (Stathakis et al., 1999). The name of the gene comes from the high levels of catecholamines of the mutant flies due to the hyperactivity of the tyrosine hydroxylase (TH) enzyme. This result showed that *Catsup* is a negative regulator of dopamine synthesis. As a result, mutants display high synaptic activity and elevated mobility (Stathakis et al., 1999; Wang et al., 2011). *Catsup* has a very broad expression pattern (almost ubiquitous) and it is localized in the endoplasmic reticulum (ER) and Golgi apparatus (Groth et al., 2013). Accordingly, *Catsup* mutant flies accumulate misfolded proteins such as Notch and APPL in the Golgi apparatus and in the ER. Probably loss of *catsup* affects the normal processing and trafficking of proteins that, in turn, increases ER stress and caspase 3 activity (Groth et al., 2013). The genetic interactions suggest that *Catsup* participates in the transport of extracellular Zn and Zn efflux from Golgi apparatus (Dechen et al., 2015). Up to now, the relation of Zn with the synthesis of dopamine and the accumulation of misfolded protein in *Catsup* mutants has not yet been established. *dZip99C* is a special case. It is classified in this group due to the results of genetic interactions (Dechen et al., 2015). However, recent research has found that it is an iron supplier and not a Zn transporter (Xiao et al., 2014). However, we cannot exclude that dZip99C is able to change Zn distribution indirectly. This would also explain the modification of Zn toxic phenotypes described in the literature (Lye et al., 2012, 2013; Dechen et al., 2015). Unfortunately, there is no functional data regarding dZip48C and dZip102B besides the information present in the FlyAtlas.

### Cellular zinc exporters

The proteins that act as exporters of Zn outside of the cell are dZnT63C (dZnT1) and dZnT33D (Figure 2). *dZnT63C* was first discovered in the transcriptomic analysis of the fly response to metal toxicity (Yepiskoposyan et al., 2006). It shares homology with human ZnT1 (25.6% identity and 43.1% similarity) and it is the major responsible protein for Zn import from the gut into the body (Wang et al., 2009) although it is also expressed in other tissues such as developing eye (Lye et al., 2012), testis or salivary glands (Wang et al., 2009). As expected from the proposed function, flies with reduced *dZnT63C* accumulated Zn in the gut and have a Zn deficiency in the rest of the fly. In agreement, these flies are also sensitive to Zn deprivation. The gut tries to compensate the loss of *dZnT63C* by downregulating *dZip42C.1* and *dZip42C.2* to reduce Zn uptake. dZnT63C is also found in the Malpighian tubules where it acts in Zn reabsorption into the body (Wang et al., 2009; Yin et al., 2017). The genetic interactions performed in the eye and in the thorax of the fly to modify Zn dyshomeostasis phenotypes (Lye et al., 2013), have classified dZnT33D as another cellular Zn exporter, although some intracellular localization seems to be present as well. However, no additional information about its cellular roles is available.

### Zinc exporters into cellular compartments

This last group comprises ZnT transporters that mediate Zinc efflux from the cytoplasm into cellular compartments but that also might retain the ability of cellular export. The members of this group are dZnT35C, dZnT41F, dZnT77C, and dZnT86D (Figure 2). *dZnT35C* was first described as a gene upregulated by cadmium and Zn in flies (Yepiskoposyan et al., 2006). More importantly, it was also identified in the transcriptomic profile of *dMTF-1* mutants (Yepiskoposyan et al., 2006) as one of the targets of this transcription factor. Moreover, dZnT35C is sufficient to modulate total Zn content in the fly body. Null mutations and ubiquitous downregulation of *dZnT35C* leads to viable individuals that are more sensitive to Zn overload (Yepiskoposyan et al., 2006; Yin et al., 2017). This phenotype is in agreement with the suggested exclusive expression of *dZnT35C* in the Malpighian tubules and its role in Zn detoxification and secretion from the body (Yepiskoposyan et al., 2006; Chi et al., 2015; Yin et al., 2017). Loss of *dZnT35C* impairs Zn detoxification and reduces, in turn, Zn tolerance. The ability of KD of *dZnT35C* to improve the eye degeneration induced by *dZip71B* (Dechen et al., 2015) also suggests neuronal expression in photoreceptor cells. Although dZnT35C has been localized in the plasma membrane, a recent and very interesting result confers *dZnT35C* a new location and a new role. *dZnT35C* is pivotal for the formation of a specialized type of lyososomal related organelles (LROs) in the Malpighian tubules that store Zn (Tejeda-Guzmán et al., 2017). These LROs might be the fly equivalent of mammalian zincosomes (Zalewski et al., 1993). The possibility to transport metals via vesicle-like structures has already been reported for mammalian ATP7A (Lutsenko et al., 2007).

*dZnT86D* is a Zn transporter located in the Golgi apparatus. Its overexpression is able to produce Zn toxic phenotypes in the eye without any further genetic modification (Dechen et al., 2015). Therefore, is a very interesting tool to analyse the effects provoked by Zn accumulation in the Golgi as well as changes in the intracellular Zn distribution. The ubiquitous downregulation is lethal and also produces thorax and rough eye phenotypes when performed in a tissue-specific manner (Lye et al., 2012, 2013). All this suggests that Zn plays fundamental roles in the Golgi. The last two dZnT genes, *dZnT77C* and *dZnT41F*, were classified in this category according to the results of the genetic interactions (Lye et al., 2012, 2013; Dechen et al., 2015). However, very little is known regarding their roles in the organismal and cellular Zn metabolism. On the one hand, dZnT77C is also localized in the plasma membrane of midgut cells and collaborates with dZnT63C in supplying Zn to the body from the fly intestine (Qin et al., 2013). As expected, ubiquitous silencing also enhances Zn toxicity and worsens phenotypes derived from *dZnT63C* KD. On the other hand, *dZnT41F* is also expressed in the Malpighian tubules (Chi et al., 2015; Yin et al., 2017). The similarities between silencing of *dZnT41F* and *dZnT63C* in the Malpighian tubules suggested that *dZnT41F* also contributes to body Zn reabsorption (Yin et al., 2017).

### Other genes

Additional genes that do not belong to the ZnT/Zip families have also been identified. The single *Drosophila TRPM* gene (*dTRPM*) is a curious example. Georgiev and collaborators found that *dTRPM* is permeable to Zn. *dTRPM* deficiency triggered a strong decrease in cell size that clearly resembles the effects of cellular Zn deficiency in control flies. Further experiments suggested that *dTRPM* regulates Zn homeostasis in a specific cellular compartment that is pivotal to growth control (Georgiev et al., 2010). Other very interesting cases are the *Drosophila* aquaporin homolog *big brain* (*bib*) and *vhaPPA1-2*, a subunit of the vacuolar-type ATPase. KD of *vhaPPA1-2* or *bib* reduced sensitivity to high dietary zinc levels. Their study showed loss of these two genes altered the correct subcellular localization of zinc transporters. The defect in this new level of control of Zn homeostasis was probably due to a failure in the endosomal recycling of proteins back to the membrane that hence reduced the uptake of Zn (Wang et al., 2014). Very recently, *kuzbanian* (*kuz*) a gene that was already described as a target of *dMTF-1* (Yepiskoposyan et al., 2006) has been found to confer tolerance to Zn when overexpressed (Le Manh et al., 2017).

The unexpected finding of abnormal Zn levels in a heterozygous *fumble* mutant (Gutiérrez et al., 2010) lead to the identification of an unknown recessive factor on the X chromosome displaying a strong influence on the organismal Zn content (Afshar et al., 2013). This mutation has been named *poco-zinc* in the unpublished observations of Fanis Missirlis'group (Tejeda-Guzmán et al., 2017). Although no definitive evidence was presented, all results point toward the *white* gene as the responsible for the *poco-zinc* phenotype. Following this initial discovery, authors realized that other mutants of genes involved in the transport of eye pigments, such as *scarlet* (*st*) or *carmine* (*cm*), also display reduced Zn levels. It seems that the collective action of most of these proteins is required for the biosynthesis of Zn storage granules in the Malpighian tubules of *Drosophila* (Tejeda-Guzmán et al., 2017) and thus to maintain systemic Zn homeostasis. Remarkably, the process of characterization of the *poco-zinc* locus has also highlighted the crucial importance of controlling the genetic background of flies in order to avoid misinterpretation of results when analyzing Zn metabolism and comparing different genotypes. Finally, in a recent publication from Norbert Perrimon's lab, a *Drosophila* cell-based RNAi-screen has allowed the identification of novel genes conferring sensitivity or protection toward Zn such as CG11897 (*red dog mine, rdog*) and some disease-related genes such as *IA-2 protein tyrosine phosphatase*, an ortholog of human PTPRN or *CG32000*, a putative ortholog of human ATP13A2 (Mohr et al., 2017).

## The role of Cu and Zn in *Drosophila* models of human neurodegenerative diseases

Cu also exerts some specific roles in the nervous system. Cu is necessary in flies and mammals for the biosynthesis of neurotransmitters (Sellami et al., 2012; Opazo et al., 2014) and thus, for brain activity. In addition, Cu modulates and even blocks the activity of receptors involved in neurotransmission such as NMDA, AMPA or GABA. It has been suggested that these synaptic roles are mediated by interplays between Cu and proteins like the amyloid precursor protein (APP), the prion protein (PrP), α-synuclein (α-syn) or neurotrophic factors. Interestingly, PrP proteins seem to be target genes of MTF-1 (Grzywacz et al., 2015). Moreover, Cu levels are altered in brains of patients suffering from AD, Huntington's Disease (HD) and PD. In AD and PD, Cu levels are overall reduced but enriched in the amyloid plaques, whereas Cu accumulates in HD brains (Fox et al., 2007; Davies et al., 2016; McAllum and Finkelstein, 2016). Cu binds directly bind to α-syn and promotes the formation of toxic oligomers (Davies et al., 2016). Similarly, Cu has also been found to facilitate amyloid beta (Aβ) deposition, formation of tau fibrills (McAllum and Finkelstein, 2016) as well as aggregation of Huntingtin (Htt) containing aberrant polyQ expansions (Fox et al., 2007). All these evidences and many more (Opazo et al., 2014; D'Ambrosi and Rossi, 2015) reveal new connections between Cu and neurodegenerative diseases. The critical role of Cu in several neurodevelopmental, neuromuscular and neurodegenerative disorders has been recently corroborated with the characterization of the cellular interactome of human ATP7A (Comstra et al., 2017). Similarly, Zn is also concentrated in glutamatergic synaptic terminals where it interacts with neurotransmitter receptors to modulate synaptic activity (Sensi et al., 2009, 2011). Although evidences about Zn levels in AD brains are contradictory, Zn metabolism is clearly disturbed in AD and several studies have demonstrated that Zn is crucial in the olimerization of Aβ. Furthermore, it has been suggested that combination of Cu and Zn dyshomeostasis increased toxicity of Aβ (Sensi et al., 2011; McAllum and Finkelstein, 2016). Mutations in ATP13A2 (*PARK9*) have been associated to PD. *ATP13A2* encodes a P5_B_-type ATPase and although its specific substrate is still not clear, some evidences point toward Zn as a very good candidate (Tsunemi and Krainc, 2014).

### Cu-related diseases

Menkes disease (MD) is the most important and best characterized neurological disorder related to Cu homeostasis. MD is a pediatric fatal metabolic syndrome accompanied with neurodevelopmental and neurodegenerative defects with a prevalence of 1:50,000 individuals (de Bie et al., 2007). MD is a recessive disorder produced by a loss of function of ATP7A. In the last 50 years, more than 370 mutations affecting ATP7A have been described. Depending on the severity of the symptoms, they have been ascribed, to different types of X-linked disorders named classical MD, occipital horn syndrome and spinal muscular atrophy (Zlatic et al., 2015). However, the mechanism of neurodegeneration is still poorly understood. Impairment of ATP7A functions induces accumulation of Cu into intestinal enterocytes but a deficiency of Cu in plasma, kidney, neurons and astrocytes due to the lack of transport of Cu into these tissues (Kaler, 2011). Mutations in ATP7A triggers several neurological phenotypes such as abnormal neuroblast migration, atrophy of gray and white matter as well as loss of neurons in the brain cortex and aberrant dendritic arborizations (Zlatic et al., 2015). The so called “oligoenzymatic hypothesis” (lack of Cu in cuproenzymes) does not explain the constellation of neurological manifestations and therefore, other proteins must be involved, too. In this sense, the existing mouse models very nicely recapitulate the human physiological and cellular defects (Lenartowicz et al., 2015) but they have failed to unveil new elements of the pathogenic mechanism. Moreover, it is still a matter of debate whether neurodegeneration in Menkes is a consequence of loss of ATP7A in neurons or of nutritional Cu. The potential of the fly will be very helpful to address particular roles of Cu in neurological disorders as well as to unveil novel neuronal specific proteins involved in Cu homeostasis and in the pathogenesis of MD.

As stated above, *DmATP7* is the fly ortholog of *ATP7A* (Southon et al., 2004). Remarkably, null alleles of *DmATP7* resemble human MD phenotypes (Norgate et al., 2006) and *DmATP7* is able to restore normal copper levels in MD patient's fibroblasts (Southon et al., 2010). All this shows a clear functional homology between human and fly proteins. Analysis of mutants shows that *DmATP7* is essential in early development as mutants never reach third instar larvae. Mutant larvae also display hypopigmentation (due to lack of *Tyr* activity) and lethargic behavior (because of compromised synthesis of neurotransmitters). Therefore, *DmATP7* plays a dual role exporting Cu from the cells and delivering it to cuproenzymes (Norgate et al., 2006). Silencing of *DmATP7* in the fly digestive tract is sufficient to induce Cu retention in the gut (Binks et al., 2010) and to reduce Cu content in the nervous system. This systemic effect triggers, in turn, neurodevelopmental Menkes-like phenotypes such as enhanced pupal lethality and smaller brain size (Bahadorani et al., 2010a). However, the small proportion of adults that successfully completed development show normal lifespan, but with hypersensitivity to oxidative insult (Bahadorani et al., 2010a) likely because lack of a functional antioxidant enzyme Cu/ZnSOD. Reduction of *DmATP*7 in different neuropeptidergic neurons had a strong impact on amidation of several neuropeptides but effects on fly behavior were more limited than anticipated (Sellami et al., 2012). *DmATP7* has recently been shown to interact with the oligomeric Golgi complex (COG) to control the development of synapsis via a novel mechanism (Comstra et al., 2017). All these results indicate that *DmATP7* plays cell and non-autonomous roles in Cu homeostasis. Moreover, survival of flies lacking *DmATP7* is improved by increasing Cu content in the food and by upregulating *dMTF-1*. This will consequently promote expression of *Ctr1B* to potentiate supply of Cu and of Mtns to reduce toxic effects of Cu accumulation in the gut (Bahadorani et al., 2010a). It is important to highlight that *Drosophila* only contains one ATP7 ortholog and therefore the fruit fly might also be an interesting organism to model Wilson's disease (WD), a human disorder caused by mutations in the ATP7B gene which produces increased intracellular Cu levels, accumulation of ROS, mitochondrial dysfunction and cell death (Bandmann et al., 2015). The fly models of MD and WD have gone one step beyond since they are being used as diagnostic tools to establish the pathogenicity of variants found in patients. The ability of given variants to improve the larval lethality of the fly null mutant helps to discriminate between pathological and control-like variants. In addition, subcellular localization of mutant forms have provided insight into the mechanism of copper dyshomeostasis in MD and WD patients (Mercer et al., 2017).

Several other neurodegenerative disorders have been shown to have an intimate relation with Cu (Ayton et al., 2013; McAllum and Finkelstein, 2016), although it is not clear if Cu is a friend or a foe. Usually WD patients manifest Parkinson-like symptoms (Bandmann et al., 2015). Therefore, it would be interesting to test the influence of Cu in PD models and whether manipulation of cellular Cu content might have a beneficial effect (Table 1). From the whole plethora of fly models (Botella et al., 2009), this has only been addressed in flies deficient for the protein Parkin (Saini et al., 2010). In this work, Cu-specific chelation by means of BCS was sufficient to increase longevity of *parkin* null mutants. Additional evidences suggested that reduction of ROS was mediating the neuroprotective effect of BCS in *parkin* deficient flies (Saini et al., 2010). It is likely that the combination of redox-activity of free Cu and the mitochondrial dysfunction in *parkin* mutant flies boosted the generation of ROS in these flies. The Cu-*parkin* relation was further corroborated by genetic interactions. Indeed, loss of *parkin* exacerbated the retinal degeneration triggered by overexpression of the Cu importer *Ctr1B* (Hua et al., 2010). Cu levels have been demonstrated to be lower in PD patients (Davies et al., 2016) although the rescue with BCS from Saini and collaborators goes in an opposite direction. Therefore, it would be of high relevance for the scientific community to study the impact of Cu biology in other PD fly models.

Modulation of Cu metabolism has also successfully improved the deleterious defects of toxic Aβ42 peptides in *Drosophila* models of AD (Lang et al., 2013; Singh et al., 2013; Table 1). Aβ proteins are the major component of plaques in AD patients. The Aβ42 isoform is more prone to aggregation due to the 2 extra aminoacids at the C-terminus (White et al., 1999). Reduction of cellular Cu content by silencing the expression of *Ctr1B* and *Ctr1C* (Lang et al., 2013), by overexpressing *DmATP7* (Lang et al., 2013) and *dMTF-1* or *MtnA* (Hua et al., 2011b) improved neurodegeneration, locomotion, longevity and oxidative stress markers. Similar effects were obtained by chemical chelation of Cu (Hua et al., 2011b; Singh et al., 2013). On the contrary, increasing cellular Cu or impairing intracellular Cu delivery worsened the phenotypes (Sanokawa-Akakura et al., 2010; Hua et al., 2011b). The work from Lang and collaborators raised a couple of interesting and paradoxical questions. First, AD phenotypes are rescued by panneuronal silencing of *Ctr1B* and *C*, genes supposed to be only expressed in fly gut and male gonads (Zhou et al., 2003; Hua et al., 2010). This indicates that better reporters to study expression patterns of these genes are needed. Second, the improvements were accompanied by increased amounts of higher-molecular weight forms of Aβ42. This result clearly questions the toxicity of the aggregates. However, similar genetic interventions in a fly model of HD expressing the mutant form of Htt (expanded polyQ tract) successfully reduced the levels of PolyQ aggregation by decreasing Cu content in flies (Xiao et al., 2013). Therefore, Cu metabolism might exert different and even opposite roles in distinct disease models. As a consequence, locomotion, survival and brain degeneration were also improved in this HD fly model (Xiao et al., 2013). These experiments revealed that Cu facilitates protein aggregation in HD. In agreement, a mutant form of Htt unable to bind Cu displayed a reduced toxicity (Xiao et al., 2013).

Other reports in the fly have identified genes with influence on neurodegenerative or neuropsychiatric diseases that resulted in new actors of Cu metabolism. Presenilins participate in the processing of Aβ proteins as they are the catalytic unit of the γ-secretase complex and their mutations underlie the majority of early-onset AD cases. Interestingly, secretase-independent roles have been reported for presenilins (Stiller et al., 2014). Silencing of *PSN*, the presenilin ortholog present in flies, in the fly gut reduced copper levels in flies and increased tolerance to excess dietary copper but also susceptibility to oxidative insult. PSN is suggested to contribute to the localization of Cu importers Ctr1A and Ctr1B in the plasma membrane (Southon et al., 2013b) but the exact action mechanism still needs to be completely elucidated. Misexpression of high molecular weight immunophilin FKBP52, which is a known interactor of the Cu chaperone *Atox1*, also modulates toxicity of Aβ42 (Sanokawa-Akakura et al., 2010). Finally, disruption of the dysbindin/BLOC-1 complex in the fly by means of *Drosophila* BLOC-1 mutants is sufficient to alter Cu metabolism suggesting that external factors might modulate schizophrenia via mechanisms that are conserved from humans to flies (Gokhale et al., 2015).

### Zn-related diseases

Zn also needs to be tightly regulated because excess or deprivation of Zn has severe health effects. Many reviews summarize the toxic consequences of Zn dyshomeostasis in humans and murine models (Fukada and Kambe, 2011; Hagmeyer et al., 2014; Kambe et al., 2014, 2015; Terrin et al., 2015). For example, Zn deficiency in the fetus affects organ growth that can result into preterm birth or even early death (Terrin et al., 2015). In mouse models, several behavioral tests have linked Zn deficiency with deficits in learning and memory or increased anxiety or depression-like disorders (Hagmeyer et al., 2014). Zn homeostasis is a pretty new field in the fly and although several new tools are being continuously implemented to further characterize the *Drosophila* Zn transporters, there are not many fly models of Zn-dependent diseases. In humans, mutations or even Single Nucleotide Polymorphisms (SNPs) in ZnT or Zip genes have been already associated to particular diseases (Fukada and Kambe, 2011; Kambe et al., 2015). For example, mutations in ZnT10 are responsible for hepatic cirrhosis that is accompanied with Parkinsonism or mutations in Zip4 triggers Acrodermatitis enteropathica. On the other hand, SNPs in ZnT8 are linked with increased susceptibility toward type I and II diabetes mellitus. From all human disorders directly related to Zn homeostasis, the Spondylocheirodysplasia-Ehlers-Danlos Syndrome-Like is the only one modeled in *Drosophila* (Xiao et al., 2014). The Ehlers-Danlos Syndrome is produced by loss of function of the human Zip13 transporter. However, the *Drosophila* ortholog *dZip99C* (CG7816) is involved in iron efflux and not in Zn transport. Loss of *dZip99C* affects iron storage by ferritin, increases cytosolic iron and, in agreement with the human counterpart, reduces collagen production. Interestingly, the fact that hZip13 but not hZip7 (a closely related gene that has been shown to import Zn) can complement *dZip99C* (Xiao et al., 2014), might suggest that the human disorder is also triggered by iron deregulation and thus, Zn defects are a secondary event.

Manipulation of Zn regulatory network has been a very successful approach to ameliorate neuronal dysfunction in *Drosophila* models of AD as well as to decipher the functional roles of Zn in this disease (Table 2). In an AD model based on overexpression of a mutant version of human Tau, results from a genetic screening showed that increasing or reducing the expression of a *dZip42C.1* and *dZnT63C* successfully modified Tau-related phenotypes in the fly (Huang et al., 2014). Reduction of Zn content by *dZnT63C* overexpression, silencing of *dZip42C.1* or chemical chelation improved longevity, brain vacuolization and retinal degeneration. Remarkably, authors proved in the fly that Tau phosphorylation was mediated by Zn and that hyperphosphorylation was a cardinal mechanism underlying toxicity. Furthermore, as Zn can directly bind Tau protein, authors mutated specific residues to suppress this interaction. This was sufficient to reduce Tau toxicity by suppressing Tau aggregation but without changing Tau phosphorylation. Thus, this work showed *in vivo* two Zn-dependent mechanisms underpinning Tau toxicity (Huang et al., 2014). In a second AD model, Zn chelation was sufficient to improve longevity, locomotion and photoreceptor degeneration in flies overexpressing Aβ42 protein. As expected, Zn supplementation exacerbated the phenotypes. This enhancement was suppressed in a special variant of Aβ42 in which Zn binding was abolished (Hua et al., 2011b). In agreement with the critical role of Zn metabolism in this model, other authors found that *dZip42C.1* level increases with age in the brain of Aβ42-expressing flies, whereas it goes down in controls. Such levels explain the accumulation of Zn in the brain of Aβ42 (Lang et al., 2012) and the toxic effects of Zn supplementation (Hua et al., 2011b). In line with these evidences, KD of *dZip42C.1* was sufficient to reduce neurodegeneration, prolong lifespan and improve memory loss as well as Aβ42 deposits in transgenic flies (Lang et al., 2012). All these results suggest that *dZip42C.1* is critically involved in the pathology of Aβ42. Paradoxically, overexpression of *dZip42C.1* also resulted in obvious memory recovery but the mechanism was not described in the manuscript.

Similar contradictory findings have been recently described in a *Drosophila* model of Friedreich's ataxia (FRDA). FRDA is the most important recessive ataxia in the Caucasian population. Impairment of transcription of the gene frataxin is the molecular cause underlying the disease (Campuzano et al., 1996). Most of the current evidences support a strong relation between frataxin and iron in several models (Kakhlon et al., 2008; Huang et al., 2009; Schmucker et al., 2011), including the fly (Soriano et al., 2013; Navarro et al., 2015; Chen et al., 2016). Moreover, genetic and chemical manipulation of iron biology was found to have a positive impact in FRDA phenotypes (Kakhlon et al., 2008; Whitnall et al., 2008; Navarro et al., 2015). Remarkably, flies displaying reduced levels of frataxin also seemed to have altered levels of other metals such as Zn and Cu. Although the contribution of other metals was already suggested by studies in human samples (Koeppen et al., 2012, 2013), this fly work was the first one showing that therapies based on such metals might be beneficial (Soriano et al., 2016). Indeed, Zn and Cu chelators improved frataxin deficiency without altering iron content. In this line, silencing either *dZip42C.1, dZip42C.2* and *dZip88E* or *dZnT35C, dZnT41F* and *dZnT63C* also improved FRDA conditions via reduction of the iron content (Table 2). All these results raise several interesting questions: Why are Cu and Zn accumulating in FRDA flies? Why does KD of genes with opposite function or acting in different cellular compartments trigger the same effect? Did they also modify the accumulation of Zn in FRDA flies? Is it possible that any of these transporters has also a mitochondrial function?

Studies of a *Drosophila* PD model took advantage of the antioxidant properties of Zn and explored the possibility to counteract the increased oxidative stress in the fly *parkin* mutant (Greene et al., 2005) by Zn supplementation. Interestingly, *parkin* mutant flies displayed altered levels of several Zn transporters such as *dZnT35C, dZnT63C*, or *foi* and had reduced Zn content in the head. Importantly, dietary Zn successfully increased eclosion frequency, survival rate and head zinc content of *parkin* mutants (Saini and Schaffner, 2010). Results showed that Zn boosted expression of Mtns and that the ROS scavenging properties of Mtns mediated the rescue mechanism. In relation to PD, *Catsup* mutants, surprisingly, have increased tolerance against paraquat induced neurotoxicity (Chaudhuri et al., 2007). This result is interesting since *Catsup* mutants display high tyrosine hydroxylase (TH) activity and therefore increased dopamine pools (Stathakis et al., 1999), whereas reduction of *TH* expression and dopamine levels rescued PD phenotypes in two other fly models of the disease (Bayersdorfer et al., 2010). Although the mechanism driven by loss of *Catsup* was not addressed in the manuscript, we can speculate that the activation of stress pathways in the mutants (Groth et al., 2013) might contribute to counteract the paraquat effect.

In the literature, the relation between Zn and fly models of human diseases is mostly based on studies about neurodegenerative disorders (Table 2). Importantly, the fly has also been useful to mimic other human diseases and expand their knowledge beyond the state of the art. A recent report showed that Zn is also a crucial mediator in the formation of urinary stones. In human, deficiency of xanthine dehydrogenase is responsible of such kidney stones (Arikyants et al., 2007). In the fly, silencing of the ortholog gene (*Xdh* also known as *ry*, CG7642) also induces the presence of small stones in the intraluminal content of Malpighian tubules. Spectrometric analysis of such stones revealed high similarity to the human ones including the presence of abundant Zn. In line with this novel perspective, reduction of the expression of three ZnT transporters (*dZnT35C, dZnT41F, dZnT63C*) and of *dZip71B* that are highly expressed in the Malpighian tubules mitigated the formation of stones as well as other phenotypes associated to loss of *Xdh* (Chi et al., 2015; Yin et al., 2017). Evidences suggest that downregulation of these genes avoid the accumulation of Zn in the lumen of the tubules. In agreement, Zn chelation by N,N,N'N'-tetrakis- (2-pyridylmethyl)ethylenediamine (TPEN) also lead to similar results (Chi et al., 2015). A link between cancer and Zn has been established throughout the processing of the morphogen hedgehog (*hh*). Zn normally inhibits fly hh autoprocessing and thus upon Zn deficiency there is an overactivation of the pathway that might contribute to pathogenesis of several cancer types (Xie et al., 2015).

## The potential of *Drosophila* for future studies

However, there are some aspects of Cu and Zn biology that still remain obscure. For example, it is of paramount importance to understand the need of so many ZnTs and Zips to regulate Zn metabolism. The number of genes performing similar functions confers an unexpected level of complexity. Furthermore, establishing the precise spatial and temporal expression pattern of each gene regulating Cu and Zn homeostasis will facilitate the interpretation of many aspects of the metal biology. Frank Schnorrer's and Hugo Bellen's labs have created two large-scale transgenic collections that allow protein visualization. The first one is based on a fosmid library of clones in which target genes are C-terminally tagged within their genomic context (Sarov et al., 2016). The second one has modified the *Minos* mediated integration cassette (MiMIC) to introduce a dominant marker and a gene-trap cassette flanked by two inverted ΦC31 *attP* sites (Venken et al., 2011). The MiMIC collection also allows the replacement of the gene-trap cassette via recombinase mediated cassette exchange (RMCE) even without the need of microinjection (Nagarkar-Jaiswal et al., 2015a,b). A similar approach combining CRISPR and RMCE has been reported (Zhang et al., 2014). The labs of Christopher Potter, Matthias Landgraf and Benjamin White have very nicely mixed both, MiMIC, and CRISPR, strategies and have generated another collection of so called Trojan-GAL4 lines (Diao et al., 2015). This strategy takes advantage of MiMIC transposons located in introns between coding exons and exchanges their content with a T2A-GAL4 cassette. Because of it, the native gene product will be cleaved and translation of GAL4 will occur as an independent protein. A perfect complement to these reporters in the possibility to perform cell-type-specific transcriptional profiling without cell isolation (Southall et al., 2013; Marshall et al., 2016). This extremely powerful tool developed in Andrea Brand's lab might help to clarify the cells/tissues expressing the different Zn and Cu transporters.

Up to now, tissue-specific analysis of Cu and Zn relies on RNAi lines. However, they are not completely effective and the residual gene activity might be sufficient to hide some important aspects of gene function. To bypass this, Simon Bullock's lab has developed a fast approach to generate CRISPR mutants by means of the UAS/GAL4 system (Port and Bullock, 2016). Finally, the possibility to perform unbiased genetic screens, which confers *Drosophila* an important advantage over other higher eukaryotic model systems, will allow to identify new genetic factors involved in the regulatory networks of Cu and Zn and modifiers of phenotypes in the fly models of human diseases.

## Author contributions

JAN and SS designed, wrote and approved the final manuscript.

### Conflict of interest statement

The authors declare that the research was conducted in the absence of any commercial or financial relationships that could be construed as a potential conflict of interest.

## Abbreviations

Aβ: A-beta
AD: Alzheimer's Disease
APP: amyloid precursor protein
BCS: bathocuproine disulfonate
BMP: Bone Morphogenetic Protein
Cu: Copper
COX: Cytochrome c Oxidase
CRISPR/CAS9: clustered regularly interspaced short palindromic repeat/ endonuclease CRISPR-associated 9
ER: Endoplasmic Reticulum
FLP: Flippase
FRDA: Friedreich's Ataxia
FRT: Flippase Recognition Target
HD: Huntington's Disease
hh: Hedgehog
Htt: Huntingtin
KD: Knockdown
KSD: Kidney Stone Disease
LROs: Lysosomal Related Organelles
MRE: metal responsive element
Mtns: Methallothioneins
PD: Parkinson's Disease
Phm: peptidylglycine-hydroxylating monooxygenase
PrP: Prion Protein
qMtn: Quadruple mutant of MtnsA-D
ROS: Reactive Oxygen Species
SNPs: Single Nucleotide Polymorphisms
TGFβ: Transforming Growth factor beta
TGN: Trans-Golgi Network
TPEN: N,N,N'N'-tetrakis- (2-pyridylmethyl)ethylenediamine
Tyr: Tyrosinase
Zn: Zinc.

## References

[B1] Afshar N. Argunhan B. Bettedi L. Szular J. Missirlis F. (2013). A recessive X-linked mutation causes a threefold reduction of total body zinc accumulation in *Drosophila melanogaster* laboratory strains. FEBS Open Bio. 3, 302–304. 10.1016/j.fob.2013.07.003 23951551 PMC3741916

[B2] Al-Momani F. A. Massadeh A. M. (2005). Effect of different heavy-metal concentrations on *Drosophila melanogaster* larval growth and development. Biol. Trace Elem. Res. 108, 271–277. 10.1385/BTER:108:1-3:271 16327078

[B3] Arikyants N. Sarkissian A. Hesse A. Eggermann T. Leumann E. Steinmann B. (2007). Xanthinuria type I: a rare cause of urolithiasis. Pediatr. Nephrol. 22, 310–314. 10.1007/s00467-006-0267-3 17115198

[B4] Atanesyan L. Günther V. Celniker S. E. Georgiev O. Schaffner W. (2011). Characterization of MtnE, the fifth metallothionein member in *Drosophila*. J. Biol. Inorg. Chem. 16, 1047–1056. 10.1007/s00775-011-0825-4 21870250

[B5] Ayton S. Lei P. Bush A. I. (2013). Metallostasis in Alzheimer's disease. Free Radic. Biol. Med. 62, 76–89. 10.1016/j.freeradbiomed.2012.10.558 23142767

[B6] Bahadorani S. Bahadorani P. Marcon E. Walker D. W. Hilliker A. J. (2010a). A *Drosophila* model of Menkes disease reveals a role for DmATP7 in copper absorption and neurodevelopment. Dis. Model. Mech. 3, 84–91. 10.1242/dmm.002642 20038716

[B7] Bahadorani S. Mukai S. Egli D. Hilliker A. J. (2010b). Overexpression of metal-responsive transcription factor (MTF-1) in *Drosophila melanogaster* ameliorates life-span reductions associated with oxidative stress and metal toxicity. Neurobiol. Aging 31, 1215–1226. 10.1016/j.neurobiolaging.2008.08.001 18775584

[B8] Balamurugan K. Egli D. Hua H. Rajaram R. Seisenbacher G. Georgiev O.. (2007). Copper homeostasis in *Drosophila* by complex interplay of import, storage and behavioral avoidance. EMBO J. 26, 1035–1044. 10.1038/sj.emboj.7601543 17290228 PMC1852831

[B9] Balamurugan K. Egli D. Selvaraj A. Zhang B. Georgiev O. Schaffner W. (2004). Metal-responsive transcription factor (MTF-1) and heavy metal stress response in *Drosophila* and mammalian cells: a functional comparison. Biol. Chem. 385, 597–603. 10.1515/BC.2004.074 15318808

[B10] Balamurugan K. Hua H. Georgiev O. Schaffner W. (2009). Mercury and cadmium trigger expression of the copper importer Ctr1B, which enables *Drosophila* to thrive on heavy metal-loaded food. Biol. Chem. 390, 109–113. 10.1515/BC.2009.020 19040355

[B11] Bandmann O. Weiss K. H. Kaler S. G. (2015). Wilson's disease and other neurological copper disorders. Lancet Neurol. 14, 103–113. 10.1016/S1474-4422(14)70190-5 25496901 PMC4336199

[B12] Bargiello T. A. Young M. W. (1984). Molecular genetics of a biological clock in *Drosophila*. Proc. Natl. Acad. Sci. U.S.A. 81, 2142–2146. 10.1073/pnas.81.7.2142 16593450 PMC345453

[B13] Bassett A. R. Liu J.-L. (2014). CRISPR/Cas9 and genome editing in *Drosophila*. J. Genet Genomics 41, 7–19. 10.1016/j.jgg.2013.12.004 24480743

[B14] Bayersdorfer F. Voigt A. Schneuwly S. Botella J. A. (2010). Dopamine-dependent neurodegeneration in *Drosophila* models of familial and sporadic Parkinson's disease. Neurobiol. Dis. 40, 113–119. 10.1016/j.nbd.2010.02.012 20211259

[B15] Binks T. Lye J. C. Camakaris J. Burke R. (2010). Tissue-specific interplay between copper uptake and efflux in *Drosophila*. J. Biol. Inorg. Chem. 15, 621–628. 10.1007/s00775-010-0629-y 20151166

[B16] Bonilla-Ramirez L. Jimenez-Del-Rio M. Velez-Pardo C. (2011). Acute and chronic metal exposure impairs locomotion activity in *Drosophila melanogaster*: a model to study Parkinsonism. Biometals 24, 1045–1057. 10.1007/s10534-011-9463-0 21594680

[B17] Bonneton F. Théodore L. Silar P. Maroni G. Wegnez M. (1996). Response of *Drosophila* metallothionein promoters to metallic, heat shock and oxidative stresses. FEBS Lett. 380, 33–38. 10.1016/0014-5793(95)01544-2 8603742

[B18] Botella J. A. Bayersdorfer F. Gmeiner F. Schneuwly S. (2009). Modelling Parkinson's disease in *Drosophila*. Neuromolecular Med. 11, 268–280. 10.1007/s12017-009-8098-6 19855946

[B19] Bouleau S. Tricoire H. (2015). *Drosophila* models of Alzheimer's disease: advances, limits, and perspectives. J. Alzheimers Dis. 45, 1015–1038. 10.3233/JAD-142802 25697708

[B20] Brand A. H. Perrimon N. (1993). Targeted gene expression as a means of altering cell fates and generating dominant phenotypes. Development 118, 401–415. 8223268 10.1242/dev.118.2.401

[B21] Burke R. Commons E. Camakaris J. (2008). Expression and localisation of the essential copper transporter DmATP7 in *Drosophila* neuronal and intestinal tissues. Int. J. Biochem. Cell Biol. 40, 1850–1860. 10.1016/j.biocel.2008.01.021 18321764

[B22] Calap-Quintana P. González-Fernández J. Sebastiá-Ortega N. Llorens J. V. Moltó M. D. (2017). *Drosophila melanogaster* models of metal-related human diseases and metal toxicity. Int. J. Mol. Sci. 18:E1456. 10.3390/ijms18071456 28684721 PMC5535947

[B23] Campuzano V. Montermini L. Moltò M. D. Pianese L. Cossée M. Cavalcanti F.. (1996). Friedreich's ataxia: autosomal recessive disease caused by an intronic GAA triplet repeat expansion. Science 271, 1423–1427. 10.1126/science.271.5254.1423 8596916

[B24] Carrasco-Rando M. Atienza-Manuel A. Martín P. Burke R. Ruiz-Gómez M. (2016). Fear-of-intimacy-mediated zinc transport controls the function of zinc-finger transcription factors involved in myogenesis. Development 143, 1948–1957. 10.1242/dev.131953 27068109

[B25] Casci I. Pandey U. B. (2015). A fruitful endeavor: modeling ALS in the fruit fly. Brain Res. 1607, 47–74. 10.1016/j.brainres.2014.09.064 25289585 PMC4385417

[B26] Chandra S. Pandey A. Chowdhuri D. K. (2015). MiRNA profiling provides insights on adverse effects of Cr(VI) in the midgut tissues of *Drosophila melanogaster*. J. Hazard. Mater. 283, 558–567. 10.1016/j.jhazmat.2014.09.054 25464296

[B27] Chaudhuri A. Bowling K. Funderburk C. Lawal H. Inamdar A. Wang Z.. (2007). Interaction of genetic and environmental factors in a *Drosophila* parkinsonism model. J. Neurosci. 27, 2457–2467. 10.1523/JNEUROSCI.4239-06.2007 17344383 PMC6672491

[B28] Chen K. Lin G. Haelterman N. A. Ho T. S. Li T. Li Z.. (2016). Loss of Frataxin induces iron toxicity, sphingolipid synthesis, and Pdk1/Mef2 activation, leading to neurodegeneration. ELife 5:e16043. 10.7554/eLife.16043 27343351 PMC4956409

[B29] Chen X. Hua H. Balamurugan K. Kong X. Zhang L. George G. N.. (2008). Copper sensing function of *Drosophila* metal-responsive transcription factor-1 is mediated by a tetranuclear Cu(I) cluster. Nucleic Acids Res. 36, 3128–3138. 10.1093/nar/gkn103 18411209 PMC2396432

[B30] Chi T. Kim M. S. Lang S. Bose N. Kahn A. Flechner L.. (2015). A *Drosophila* model identifies a critical role for zinc in mineralization for kidney stone disease. PLoS ONE 10:e0124150. 10.1371/journal.pone.0124150 25970330 PMC4430225

[B31] Choi S. Bird A. J. (2014). Zinc'ing sensibly: controlling zinc homeostasis at the transcriptional level. Metallomics 6, 1198–1215. 10.1039/c4mt00064a 24722954

[B32] Clark I. E. Dodson M. W. Jiang C. Cao J. H. Huh J. R. Seol J. H.. (2006). *Drosophila* pink1 is required for mitochondrial function and interacts genetically with parkin. Nature 441, 1162–1166. 10.1038/nature04779 16672981

[B33] Comstra H. S. McArthy J. Rudin-Rush S. Hartwig C. Gokhale A. Zlatic S. A.. (2017). The interactome of the copper transporter ATP7A belongs to a network of neurodevelopmental and neurodegeneration factors. ELife 6:e24722. 10.7554/eLife.24722 28355134 PMC5400511

[B34] D'Ambrosi N. Rossi L. (2015). Copper at synapse: release, binding and modulation of neurotransmission. Neurochem. Int. 90, 36–45. 10.1016/j.neuint.2015.07.006 26187063

[B35] de Bie P. Muller P. Wijmenga C. Klomp L. W. (2007). Molecular pathogenesis of Wilson and Menkes disease: correlation of mutations with molecular defects and disease phenotypes. J. Med. Genet. 44, 673–688. 10.1136/jmg.2007.052746 17717039 PMC2752173

[B36] Davies K. M. Mercer J. F. B. Chen N. Double K. L. (2016). Copper dyshomoeostasis in Parkinson's disease: implications for pathogenesis and indications for novel therapeutics. Clin. Sci. (Lond) 130, 565–574. 10.1042/CS20150153 26957644

[B37] Dechen K. Richards C. D. Lye J. C. Hwang J. E. C. Burke R. (2015). Compartmentalized zinc deficiency and toxicities caused by ZnT and Zip gene over expression result in specific phenotypes in *Drosophila*. Int. J. Biochem. Cell Biol. 60, 23–33. 10.1016/j.biocel.2014.12.017 25562517

[B38] Diao F. Ironfield H. Luan H. Diao F. Shropshire W. C. Ewer J.. (2015). Plug-and-play genetic access to *Drosophila* cell types using exchangeable exon cassettes. Cell Rep. 10, 1410–1421. 10.1016/j.celrep.2015.01.059 25732830 PMC4373654

[B39] Dietzl G. Chen D. Schnorrer F. Su K.-C. Barinova Y. Fellner M.. (2007). A genome-wide transgenic RNAi library for conditional gene inactivation in *Drosophila*. Nature 448, 151–156. 10.1038/nature05954 17625558

[B40] Duffy J. B. (2002). GAL4 system in *Drosophila*: a fly geneticist's Swiss army knife. Genesis 34, 1–15. 10.1002/gene.10150 12324939

[B41] Durliat M. Bonneton F. Boissonneau E. André M. Wegnez M. (1995). Expression of metallothionein genes during the post-embryonic development of *Drosophila melanogaster*. Biometals 8, 339–351. 10.1007/BF00141608 7580054

[B42] Egli D. Domènech J. Selvaraj A. Balamurugan K. Hua H. Capdevila M.. (2006a). The four members of the *Drosophila* metallothionein family exhibit distinct yet overlapping roles in heavy metal homeostasis and detoxification. Genes Cells 11, 647–658. 10.1111/j.1365-2443.2006.00971.x 16716195

[B43] Egli D. Selvaraj A. Yepiskoposyan H. Zhang B. Hafen E. Georgiev O.. (2003). Knockout of 'metal-responsive transcription factor' MTF-1 in *Drosophila* by homologous recombination reveals its central role in heavy metal homeostasis. EMBO J. 22, 100–108. 10.1093/emboj/cdg012 12505988 PMC140060

[B44] Egli D. Yepiskoposyan H. Selvaraj A. Balamurugan K. Rajaram R. Simons A.. (2006b). A family knockout of all four *Drosophila* metallothioneins reveals a central role in copper homeostasis and detoxification. Mol. Cell. Biol. 26, 2286–2296. 10.1128/MCB.26.6.2286-2296.2006 16508004 PMC1430275

[B45] Esposito G. Vos M. Vilain S. Swerts J. De Sousa Valadas J. van Meensel S.. (2013). Aconitase causes iron toxicity in *Drosophila* pink1 mutants. PLoS Genet. 9:e1003478. 10.1371/journal.pgen.1003478 23637640 PMC3636082

[B46] Feany M. B. Bender W. W. (2000). A *Drosophila* model of Parkinson's disease. Nature 404, 394–398. 10.1038/35006074 10746727

[B47] Folwell J. L. Barton C. H. Shepherd D. (2006). Immunolocalisation of the *D. melanogaster* Nramp homologue Malvolio to gut and Malpighian tubules provides evidence that Malvolio and Nramp2 are orthologous. J. Exp. Biol. 209, 1988–1995. 10.1242/jeb.02193 16651563

[B48] Fox J. H. Kama J. A. Lieberman G. Chopra R. Dorsey K. Chopra V.. (2007). Mechanisms of copper ion mediated Huntington's disease progression. PLoS ONE 2:e334. 10.1371/journal.pone.0000334 17396163 PMC1828629

[B49] Fukada T. Kambe T. (2011). Molecular and genetic features of zinc transporters in physiology and pathogenesis. Metallomics 3, 662–674. 10.1039/c1mt00011j 21566827

[B50] Georgiev P. Okkenhaug H. Drews A. Wright D. Lambert S. Flick M.. (2010). TRPM channels mediate zinc homeostasis and cellular growth during *Drosophila* larval development. Cell Metab. 12, 386–397. 10.1016/j.cmet.2010.08.012 20889130

[B51] Gokhale A. Vrailas-Mortimer A. Larimore J. Comstra H. S. Zlatic S. A. Werner E.. (2015). Neuronal copper homeostasis susceptibility by genetic defects in dysbindin, a schizophrenia susceptibility factor. Hum. Mol. Genet. 24, 5512–5523. 10.1093/hmg/ddv282 26199316 PMC4572075

[B52] Golic K. G. (1991). Site-specific recombination between homologous chromosomes in *Drosophila*. Science 252, 958–961. 10.1126/science.2035025 2035025

[B53] Graveley B. R. Brooks A. N. Carlson J. W. Duff M. O. Landolin J. M. Yang L.. (2011). The developmental transcriptome of *Drosophila melanogaster*. Nature 471, 473–479. 10.1038/nature09715 21179090 PMC3075879

[B54] Greene J. C. Whitworth A. J. Andrews L. A. Parker T. J. Pallanck L. J. (2005). Genetic and genomic studies of *Drosophila* parkin mutants implicate oxidative stress and innate immune responses in pathogenesis. Hum. Mol. Genet. 14, 799–811. 10.1093/hmg/ddi074 15689351

[B55] Greene J. C. Whitworth A. J. Kuo I. Andrews L. A. Feany M. B. Pallanck L. J. (2003). Mitochondrial pathology and apoptotic muscle degeneration in *Drosophila* parkin mutants. Proc. Natl. Acad. Sci. U.S.A. 100, 4078–4083. 10.1073/pnas.0737556100 12642658 PMC153051

[B56] Groth C. Sasamura T. Khanna M. R. Whitley M. Fortini M. E. (2013). Protein trafficking abnormalities in *Drosophila* tissues with impaired activity of the ZIP7 zinc transporter Catsup. Development 140, 3018–3027. 10.1242/dev.088336 23785054 PMC3699284

[B57] Grubman A. White A. R. (2014). Copper as a key regulator of cell signalling pathways. Expert Rev. Mol. Med. 16:e11. 10.1017/erm.2014.11 24849048

[B58] Grzywacz A. Gdula-Argasinska J. Muszynska B. Tyszka-Czochara M. Librowski T. Opoka W. (2015). Metal responsive transcription factor 1 (MTF-1) regulates zinc dependent cellular processes at the molecular level. Acta Biochim. Pol. 62, 491–498. 10.18388/abp.2015_1038 26336656

[B59] Guan D. Mo F. Han Y. Gu W. Zhang M. (2015). Digital gene expression profiling (DGE) of cadmium-treated *Drosophila melanogaster*. Environ. Toxicol. Pharmacol. 39, 300–306. 10.1016/j.etap.2014.11.024 25543212

[B60] Guirola M. Naranjo Y. Capdevila M. Atrian S. (2011). Comparative genomics analysis of metallothioneins in twelve *Drosophila* species. J. Inorg. Biochem. 105, 1050–1059. 10.1016/j.jinorgbio.2011.05.004 21726767

[B61] Günes C. Heuchel R. Georgiev O. Müller K. H. Lichtlen P. Blüthmann H.. (1998). Embryonic lethality and liver degeneration in mice lacking the metal-responsive transcriptional activator MTF-1. EMBO J. 17, 2846–2854. 10.1093/emboj/17.10.2846 9582278 PMC1170625

[B62] Günther V. Lindert U. Schaffner W. (2012a). The taste of heavy metals: gene regulation by MTF-1. Biochim. Biophys. Acta 1823, 1416–1425. 10.1016/j.bbamcr.2012.01.005 22289350

[B63] Gunther V. Waldvogel D. Nosswitz M. Georgiev O. Schaffner W. (2012b). Dissection of *Drosophila* MTF-1 reveals a domain for differential target gene activation upon copper overload vs. copper starvation. Int. J. Biochem. Cell Biol. 44, 404–411. 10.1016/j.biocel.2011.11.016 22138226

[B64] Gutiérrez L. Sabaratnam N. Aktar R. Bettedi L. Mandilaras K. Missirlis F. (2010). Zinc accumulation in heterozygous mutants of fumble, the pantothenate kinase homologue of *Drosophila*. FEBS Lett. 584, 2942–2946. 10.1016/j.febslet.2010.05.029 20493851

[B65] Hagmeyer S. Haderspeck J. C. Grabrucker A. M. (2014). Behavioral impairments in animal models for zinc deficiency. Front. Behav. Neurosci. 8:443. 10.3389/fnbeh.2014.00443 25610379 PMC4285094

[B66] Harris E. D. (2001). Copper homeostasis: the role of cellular transporters. Nutr. Rev. 59, 281–285. 10.1111/j.1753-4887.2001.tb07017.x 11570430

[B67] He T. Hirsch H. V. B. Ruden D. M. Lnenicka G. A. (2009). Chronic lead exposure alters presynaptic calcium regulation and synaptic facilitation in *Drosophila* larvae. Neurotoxicology 30, 777–784. 10.1016/j.neuro.2009.08.007 19732793 PMC2796506

[B68] Helfand S. L. Rogina B. (2003). Genetics of aging in the fruit fly, *Drosophila melanogaster*. Annu. Rev. Genet. 37, 329–348. 10.1146/annurev.genet.37.040103.095211 14616064

[B69] Hua H. Georgiev O. Schaffner W. Steiger D. (2010). Human copper transporter Ctr1 is functional in *Drosophila*, revealing a high degree of conservation between mammals and insects. J. Biol. Inorg. Chem. 15, 107–113. 10.1007/s00775-009-0599-0 19856191

[B70] Hua H. Günther V. Georgiev O. Schaffner W. (2011a). Distorted copper homeostasis with decreased sensitivity to cisplatin upon chaperone Atox1 deletion in *Drosophila*. Biometals 24, 445–453. 10.1007/s10534-011-9438-1 21465178

[B71] Hua H. Münter L. Harmeier A. Georgiev O. Multhaup G. Schaffner W. (2011b). Toxicity of Alzheimer's disease-associated Abeta peptide is ameliorated in a *Drosophila* model by tight control of zinc and copper availability. Biol. Chem. 392, 919–926. 10.1515/BC.2011.084 21801085

[B72] Huang M. L. Becker E. M. Whitnall M. Suryo Rahmanto Y. Ponka P. Richardson D. R. (2009). Elucidation of the mechanism of mitochondrial iron loading in Friedreich's ataxia by analysis of a mouse mutant. Proc. Natl. Acad. Sci. U.S.A. 106, 16381–16386. 10.1073/pnas.0906784106 19805308 PMC2752539

[B73] Huang Y. Wu Z. Cao Y. Lang M. Lu B. Zhou B. (2014). Zinc binding directly regulates tau toxicity independent of tau hyperphosphorylation. Cell Rep. 8, 831–842. 10.1016/j.celrep.2014.06.047 25066125 PMC4306234

[B74] Hwang J. E. de Bruyne M. Warr C. G. Burke R. (2014). Copper overload and deficiency both adversely affect the central nervous system of *Drosophila*. Metallomics 6, 2223–2229. 10.1039/C4MT00140K 25322772

[B75] Jürgens G. Wieschaus E. Nüsslein-Volhard C. Kluding H. (1984). Mutations affecting the pattern of the larval cuticle in *Drosophila* melanogaster: II. Zygotic loci on the third chromosome. Wilhelm Roux Arch. Dev. Biol. 193, 283–295. 10.1007/BF00848157 28305338

[B76] Kakhlon O. Manning H. Breuer W. Melamed-Book N. Lu C. Cortopassi G.. (2008). Cell functions impaired by frataxin deficiency are restored by drug-mediated iron relocation. Blood 112, 5219–5227. 10.1182/blood-2008-06-161919 18796625

[B77] Kaler S. G. (2011). ATP7A-related copper transport diseases-emerging concepts and future trends. Nat. Rev. Neurol. 7, 15–29. 10.1038/nrneurol.2010.180 21221114 PMC4214867

[B78] Kambe T. Hashimoto A. Fujimoto S. (2014). Current understanding of ZIP and ZnT zinc transporters in human health and diseases. Cell. Mol. Life. Sci. 71, 3281–3295. 10.1007/s00018-014-1617-0 24710731 PMC11113243

[B79] Kambe T. Tsuji T. Hashimoto A. Itsumura N. (2015). The physiological, biochemical, and molecular roles of zinc transporters in zinc homeostasis and metabolism. Physiol. Rev. 95, 749–784. 10.1152/physrev.00035.2014 26084690

[B80] Kelly E. J. Quaife C. J. Froelick G. J. Palmiter R. D. (1996). Metallothionein I and II protect against zinc deficiency and zinc toxicity in mice. J. Nutr. 126, 1782–1790. 8683339 10.1093/jn/126.7.1782

[B81] Kim B.-E. Turski M. L. Nose Y. Casad M. Rockman H. A. Thiele D. J. (2010). Cardiac copper deficiency activates a systemic signaling mechanism that communicates with the copper acquisition and storage organs. Cell Metab. 11, 353–363. 10.1016/j.cmet.2010.04.003 20444417 PMC2901851

[B82] Kirby K. Jensen L. T. Binnington J. Hilliker A. J. Ulloa J. Culotta V. C.. (2008). Instability of superoxide dismutase 1 of *Drosophila* in mutants deficient for its cognate copper chaperone. J. Biol. Chem. 283, 35393–35401. 10.1074/jbc.M807131200 18948262 PMC2602909

[B83] Koeppen A. H. Kuntzsch E. C. Bjork S. T. Ramirez R. L. Mazurkiewicz J. E. Feustel P. J. (2013). Friedreich ataxia: metal dysmetabolism in dorsal root ganglia. Acta Neuropathol. Commun. 1:26. 10.1186/2051-5960-1-26 24252376 PMC3893523

[B84] Koeppen A. H. Ramirez R. L. Yu D. Collins S. E. Qian J. Parsons P. J.. (2012). Friedreich's ataxia causes redistribution of iron, copper, and zinc in the dentate nucleus. Cerebellum 11, 845–860. 10.1007/s12311-012-0383-5 22562713 PMC3497958

[B85] Konopka R. J. Benzer S. (1971). Clock mutants of *Drosophila melanogaster*. Proc. Natl. Acad. Sci. U.S.A. 68, 2112–2116. 10.1073/pnas.68.9.2112 5002428 PMC389363

[B86] Krezel A. Maret W. (2017). The functions of metamorphic metallothioneins in zinc and copper metabolism. Int. J. Mol. Sci. 18:1237. 10.3390/ijms18061237 28598392 PMC5486060

[B87] Lang M. Fan Q. Wang L. Zheng Y. Xiao G. Wang X.. (2013). Inhibition of human high-affinity copper importer Ctr1 orthologous in the nervous system of *Drosophila* ameliorates Abeta42-induced Alzheimer's disease-like symptoms. Neurobiol. Aging 34, 2604–2612. 10.1016/j.neurobiolaging.2013.05.029 23827522 PMC3770863

[B88] Lang M. Wang L. Fan Q. Xiao G. Wang X. Zhong Y.. (2012). Genetic inhibition of solute-linked carrier 39 family transporter 1 ameliorates abeta pathology in a *Drosophila* model of Alzheimer's disease. PLoS Genet. 8:e1002683. 10.1371/journal.pgen.1002683 22570624 PMC3343105

[B89] Laukens D. Waeytens A. De Bleser P. Cuvelier C. De Vos M. (2009). Human metallothionein expression under normal and pathological conditions: mechanisms of gene regulation based on in silico promoter analysis. Crit. Rev. Eukaryot. Gene Expr. 19, 301–317. 10.1615/CritRevEukarGeneExpr.v19.i4.40 19817707

[B90] Le Manh H. Guio L. Merenciano M. Rovira Q. Barrón M. G. González J. (2017). Natural and laboratory mutations in kuzbanian are associated with zinc stress phenotypes in *Drosophila melanogaster*. Sci. Rep. 7:42663. 10.1038/srep42663 28218276 PMC5316978

[B91] Lenartowicz M. Krzeptowski W. Lipinski P. Grzmil P. Starzynski R. Pierzchała O.. (2015). Mottled mice and non-mammalian models of menkes disease. Front. Mol. Neurosci. 8:72. 10.3389/fnmol.2015.00072 26732058 PMC4684000

[B92] Letelier M. E. Faúndez M. Jara-Sandoval J. Molina-Berríos A. Cortés-Troncoso J. Aracena-Parks P.. (2009). Mechanisms underlying the inhibition of the cytochrome P450 system by copper ions. J. Appl. Toxicol. 29, 695–702. 10.1002/jat.1460 19629952

[B93] Lichten L. A. Cousins R. J. (2009). Mammalian zinc transporters: nutritional and physiologic regulation. Annu. Rev. Nutr. 29, 153–176. 10.1146/annurev-nutr-033009-083312 19400752

[B94] Liu B. Moloney A. Meehan S. Morris K. Thomas S. E. Serpell L. C.. (2011). Iron promotes the toxicity of amyloid beta peptide by impeding its ordered aggregation. J. Biol. Chem. 286, 4248–4256. 10.1074/jbc.M110.158980 21147772 PMC3039358

[B95] Liuzzi J. P. Guo L. Yoo C. Stewart T. S. (2014). Zinc and autophagy. Biometals 27, 1087–1096. 10.1007/s10534-014-9773-0 25012760 PMC4224969

[B96] Lutsenko S. Barnes N. L. Bartee M. Y. Dmitriev O. Y. (2007). Function and regulation of human copper-transporting ATPases. Physiol. Rev. 87, 1011–1046. 10.1152/physrev.00004.2006 17615395

[B97] Lye J. C. Hwang J. E. C. Paterson D. de Jonge M. D. Howard D. L. Burke R. (2011). Detection of genetically altered copper levels in *Drosophila* tissues by synchrotron x-ray fluorescence microscopy. PLoS ONE 6:e26867. 10.1371/journal.pone.0026867 22053217 PMC3203902

[B98] Lye J. C. Richards C. D. Dechen K. Paterson D. de Jonge M. D. Howard D. L.. (2012). Systematic functional characterization of putative zinc transport genes and identification of zinc toxicosis phenotypes in *Drosophila melanogaster*. J. Exp. Biol. 215, 3254–3265. 10.1242/jeb.069260 22693027

[B99] Lye J. C. Richards C. D. Dechen K. Warr C. G. Burke R. (2013). *In vivo* zinc toxicity phenotypes provide a sensitized background that suggests zinc transport activities for most of the *Drosophila* Zip and ZnT genes. J. Biol. Inorg. Chem. 18, 323–332. 10.1007/s00775-013-0976-6 23322169

[B100] Madsen E. Gitlin J. D. (2007). Copper and iron disorders of the brain. Annu. Rev. Neurosci. 30, 317–337. 10.1146/annurev.neuro.30.051606.094232 17367269

[B101] Mandilaras K. Pathmanathan T. Missirlis F. (2013). Iron absorption in *Drosophila melanogaster*. Nutrients 5, 1622–1647. 10.3390/nu5051622 23686013 PMC3708341

[B102] Maroni G. Lastowski-Perry D. Otto E. Watson D. (1986). Effects of heavy metals on *Drosophila* larvae and a metallothionein cDNA. Environ. Health Perspect. 65, 107–116. 3086075 10.1289/ehp.8665107 PMC1474713

[B103] Marshall O. J. Southall T. D. Cheetham S. W. Brand A. H. (2016). Cell-type-specific profiling of protein-DNA interactions without cell isolation using targeted DamID with next-generation sequencing. Nat. Protoc. 11, 1586–1598. 10.1038/nprot.2016.084 27490632 PMC7032955

[B104] Masters B. A. Kelly E. J. Quaife C. J. Brinster R. L. Palmiter R. D. (1994). Targeted disruption of metallothionein I and II genes increases sensitivity to cadmium. Proc. Natl. Acad. Sci. U.S.A. 91, 584–588. 10.1073/pnas.91.2.584 8290567 PMC42993

[B105] Mathews W. R. Ong D. Milutinovich A. B. Van Doren M. (2006). Zinc transport activity of Fear of Intimacy is essential for proper gonad morphogenesis and DE-cadherin expression. Development 133, 1143–1153. 10.1242/dev.02256 16481356

[B106] Mathews W. R. Wang F. Eide D. J. van Doren M. (2005). Drosophila fear of intimacy encodes a Zrt/IRT-like protein (ZIP) family zinc transporter functionally related to mammalian ZIP proteins. J. Biol. Chem. 280, 787–795. 10.1074/jbc.M411308200 15509557

[B107] McAllum E. J. Finkelstein D. I. (2016). Metals in Alzheimer's and Parkinson's disease: relevance to dementia with lewy bodies. J. Mol. Neurosci. 60, 279–288. 10.1007/s12031-016-0809-5 27498879

[B108] Mercer S. W. La Fontaine S. Warr C. G. Burke R. (2016). Reduced glutathione biosynthesis in *Drosophila melanogaster* causes neuronal defects linked to copper deficiency. J. Neurochem. 137, 360–370. 10.1111/jnc.13567 26851457

[B109] Mercer S. W. Wang J. Burke R. (2017). *In vivo* modeling of the pathogenic effect of copper transporter mutations that cause menkes and wilson diseases, motor neuropathy, and susceptibility to Alzheimer's disease. J. Biol. Chem. 292, 4113–4122. 10.1074/jbc.M116.756163 28119449 PMC5354492

[B110] Meyer S. Schulz J. Jeibmann A. Taleshi M. S. Ebert F. Francesconi K. A.. (2014). Arsenic-containing hydrocarbons are toxic in the *in vivo* model *Drosophila melanogaster*. Metallomics 6, 2010–2014. 10.1039/C4MT00249K 25292248

[B111] M Fetherolf M. Boyd S. D. Winkler D. D. Winge D. R. (2017). Oxygen-dependent activation of Cu, Zn-superoxide dismutase-1. Metallomics 9, 1047–1059. 10.1039/C6MT00298F 28686251

[B112] Mohr S. E. Rudd K. Hu Y. Song W. R. Gilly Q. Buckner M.. (2017). Zinc detoxification: a functional genomics and transcriptomics analysis in *Drosophila melanogaster* cultured cells. G3 (Bethesda, MD.). [Epub ahead of print]. 10.1534/g3.117.300447 29223976 PMC5919732

[B113] Molnar J. Ujfaludi Z. Fong S. F. T. Bollinger J. A. Waro G. Fogelgren B.. (2005). *Drosophila* lysyl oxidases Dmloxl-1 and Dmloxl-2 are differentially expressed and the active DmLOXL-1 influences gene expression and development. J. Biol. Chem. 280, 22977–22985. 10.1074/jbc.M503006200 15811848

[B114] Monastirioti M. Linn C. E. White K. (1996). Characterization of *Drosophila* tyramine beta-hydroxylase gene and isolation of mutant flies lacking octopamine. J. Neurosci. 16, 3900–3911. 8656284 10.1523/JNEUROSCI.16-12-03900.1996 PMC6578608

[B115] Morgan T. H. (1910). Sex limited inheritance in *Drosophila*. Science 32, 120–122. 10.1126/science.32.812.120 17759620

[B116] Mummery-Widmer J. L. Yamazaki M. Stoeger T. Novatchkova M. Bhalerao S. Chen D.. (2009). Genome-wide analysis of Notch signalling in *Drosophila* by transgenic RNAi. Nature 458, 987–992. 10.1038/nature07936 19363474 PMC2988197

[B117] Myers E. W. Sutton G. G. Delcher A. L. Dew I. M. Fasulo D. P. Flanigan M. J.. (2000). A whole-genome assembly of *Drosophila*. Science 287, 2196–2204. 10.1126/science.287.5461.2196 10731133

[B118] Nagarkar-Jaiswal S. DeLuca S. Z. Lee P.-T. Lin W.-W. Pan H. Zuo Z.. (2015a). A genetic toolkit for tagging intronic MiMIC containing genes. ELife 4:e08469. 10.7554/eLife.08469 26102525 PMC4499919

[B119] Nagarkar-Jaiswal S. Lee P.-T. Campbell M. E. Chen K. Anguiano-Zarate S. Gutierrez M. C.. (2015b). A library of MiMICs allows tagging of genes and reversible, spatial and temporal knockdown of proteins in *Drosophila*. ELife 4:e05338. 10.7554/eLife.05338 25824290 PMC4379497

[B120] Navarro J. A. Botella J. A. Metzendorf C. Lind M. I. Schneuwly S. (2015). Mitoferrin modulates iron toxicity in a *Drosophila* model of Friedreich's ataxia. Free Radic. Biol. Med. 85, 71–82. 10.1016/j.freeradbiomed.2015.03.014 25841783

[B121] Nevitt T. Ohrvik H. Thiele D. J. (2012). Charting the travels of copper in eukaryotes from yeast to mammals. Biochim. Biophys. Acta 1823, 1580–1593. 10.1016/j.bbamcr.2012.02.011 22387373 PMC3392525

[B122] Ni J.-Q. Liu L.-P. Binari R. Hardy R. Shim H.-S. Cavallaro A.. (2009). A *Drosophila* resource of transgenic RNAi lines for neurogenetics. Genetics 182, 1089–1100. 10.1534/genetics.109.103630 19487563 PMC2728850

[B123] Niehoff A.-C. Bauer O. B. Kröger S. Fingerhut S. Schulz J. Meyer S.. (2015). Quantitative bioimaging to investigate the uptake of mercury species in *Drosophila melanogaster*. Anal. Chem. 87, 10392–10396. 10.1021/acs.analchem.5b02500 26424032

[B124] Norgate M. Lee E. Southon A. Farlow A. Batterham P. Camakaris J.. (2006). Essential roles in development and pigmentation for the *Drosophila* copper transporter DmATP7. Mol. Biol. Cell 17, 475–484. 10.1091/mbc.E05-06-0492 16251357 PMC1345683

[B125] Norgate M. Southon A. Greenough M. Cater M. Farlow A. Batterham P.. (2010). Syntaxin 5 is required for copper homeostasis in *Drosophila* and mammals. PLoS ONE 5:e14303. 10.1371/journal.pone.0014303 21188142 PMC3004795

[B126] Norgate M. Southon A. Zou S. Zhan M. Sun Y. Batterham P.. (2007). Copper homeostasis gene discovery in *Drosophila melanogaster*. Biometals 20, 683–697. 10.1007/s10534-006-9075-2 17216353

[B127] Opazo C. M. Greenough M. A. Bush A. I. (2014). Copper: from neurotransmission to neuroproteostasis. Front. Aging Neurosci. 6:143. 10.3389/fnagi.2014.00143 25071552 PMC4080678

[B128] Ortiz J. G. M. Opoka R. Kane D. Cartwright I. L. (2009). Investigating arsenic susceptibility from a genetic perspective in *Drosophila* reveals a key role for glutathione synthetase. Toxicol. Sci. 107, 416–426. 10.1093/toxsci/kfn192 18779381 PMC2639754

[B129] Osredkar J. (2011). Copper and Zinc, biological role and significance of copper/zinc imbalance. J. Clin. Toxicol. S3:001 10.4172/2161-0495.S3-001

[B130] Ott S. Dziadulewicz N. Crowther D. C. (2015). Iron is a specific cofactor for distinct oxidation- and aggregation-dependent Abeta toxicity mechanisms in a *Drosophila* model. Dis. Model. Mech. 8, 657–667. 10.1242/dmm.019042 26035384 PMC4486857

[B131] Palmiter R. D. (1998). The elusive function of metallothioneins. Proc. Natl. Acad. Sci. U.S.A. 95, 8428–8430. 10.1073/pnas.95.15.8428 9671693 PMC33872

[B132] Palumaa P. (2013). Copper chaperones. The concept of conformational control in the metabolism of copper. FEBS Lett. 587, 1902–1910. 10.1016/j.febslet.2013.05.019 23684646

[B133] Pérez-Rafael S. Kurz A. Guirola M. Capdevila M. Palacios O. Atrian S. (2012). Is MtnE, the fifth *Drosophila* metallothionein, functionally distinct from the other members of this polymorphic protein family? Metallomics 4, 342–349. 10.1039/c2mt00182a 22370740

[B134] Phillips J. P. Campbell S. D. Michaud D. Charbonneau M. Hilliker A. J. (1989). Null mutation of copper/zinc superoxide dismutase in *Drosophila* confers hypersensitivity to paraquat and reduced longevity. Proc. Natl. Acad. Sci. U.S.A. 86, 2761–2765. 10.1073/pnas.86.8.2761 2539600 PMC286998

[B135] Pielage J. Kippert A. Zhu M. Klämbt C. (2004). The *Drosophila* transmembrane protein Fear-of-intimacy controls glial cell migration. Dev. Biol. 275, 245–257. 10.1016/j.ydbio.2004.07.039 15464587

[B136] Porcelli D. Oliva M. Duchi S. Latorre D. Cavaliere V. Barsanti P.. (2010). Genetic, functional and evolutionary characterization of scox, the *Drosophila melanogaster* ortholog of the human SCO1 gene. Mitochondrion 10, 433–448. 10.1016/j.mito.2010.04.002 20388558

[B137] Port F. Bullock S. L. (2016). Augmenting CRISPR applications in *Drosophila* with tRNA-flanked sgRNAs. Nat. Methods 13, 852–854. 10.1038/nmeth.3972 27595403 PMC5215823

[B138] Poujois A. Devedjian J.-C. Moreau C. Devos D. Chaine P. Woimant F.. (2016). Bioavailable trace metals in neurological diseases. Curr. Treat. Options Neurol. 18:46. 10.1007/s11940-016-0426-1 27682263

[B139] Poulson D. F. Bowen V. T. Hilse R. M. Rubinson A. C. (1952). The copper metabolism of *Drosophila*. Proc. Natl. Acad. Sci. U.S.A. 38, 912–921. 10.1073/pnas.38.10.912 16589200 PMC1063680

[B140] Qin Q. Wang X. Zhou B. (2013). Functional studies of *Drosophila* zinc transporters reveal the mechanism for dietary zinc absorption and regulation. BMC Biol. 11:101. 10.1186/1741-7007-11-101 24063361 PMC4015762

[B141] Raudenska M. Gumulec J. Podlaha O. Sztalmachova M. Babula P. Eckschlager T.. (2014). Metallothionein polymorphisms in pathological processes. Metallomics 6, 55–68. 10.1039/C3MT00132F 24068159

[B142] Reddy P. Zehring W. A. Wheeler D. A. Pirrotta V. Hadfield C. Hall J. C.. (1984). Molecular analysis of the period locus in *Drosophila melanogaster* and identification of a transcript involved in biological rhythms. Cell 38, 701–710. 10.1016/0092-8674(84)90265-4 6435882

[B143] Reiter L. T. Potocki L. Chien S. Gribskov M. Bier E. (2001). A systematic analysis of human disease-associated gene sequences in *Drosophila melanogaster*. Genome Res. 11, 1114–1125. 10.1101/gr.169101 11381037 PMC311089

[B144] Renton A. E. Chiò A. Traynor B. J. (2014). State of play in amyotrophic lateral sclerosis genetics. Nat. Neurosci. 17, 17–23. 10.1038/nn.3584 24369373 PMC4544832

[B145] Riabinina O. Luginbuhl D. Marr E. Liu S. Wu M. N. Luo L.. (2015). Improved and expanded Q-system reagents for genetic manipulations. Nat. Methods 12, 219–222. 10.1038/nmeth.3250 25581800 PMC4344399

[B146] Richards C. D. Burke R. (2015). Local and systemic effects of targeted zinc redistribution in *Drosophila* neuronal and gastrointestinal tissues. Biometals 28, 967–974. 10.1007/s10534-015-9881-5 26411574

[B147] Richards C. D. Warr C. G. Burke R. (2015). A role for dZIP89B in *Drosophila* dietary zinc uptake reveals additional complexity in the zinc absorption process. Int. J. Biochem. Cell Biol. 69, 11–19. 10.1016/j.biocel.2015.10.004 26545796

[B148] Richards C. D. Warr C. G. Burke R. (2017). A role for the *Drosophila* zinc transporter Zip88E in protecting against dietary zinc toxicity. PLoS ONE 12:e0181237. 10.1371/journal.pone.0181237 28704512 PMC5509326

[B149] Rival T. Page R. M. Chandraratna D. S. Sendall T. J. Ryder E. Liu B.. (2009). Fenton chemistry and oxidative stress mediate the toxicity of the beta-amyloid peptide in a *Drosophila* model of Alzheimer's disease. Eur. J. Neurosci. 29, 1335–1347. 10.1111/j.1460-9568.2009.06701.x 19519625 PMC2777252

[B150] Rotilio G. Carrì M. T. Rossi L. Ciriolo M. R. (2000). Copper-dependent oxidative stress and neurodegeneration. IUBMB Life 50, 309–314. 10.1080/15216540051081074 11327325

[B151] Rovenko B. M. Perkhulyn N. V. Lushchak O. V. Storey J. M. Storey K. B. Lushchak V. I. (2014). Molybdate partly mimics insulin-promoted metabolic effects in *Drosophila melanogaster*. Comp. Biochem. Physiol. Toxicol. Pharmacol. 165, 76–82. 10.1016/j.cbpc.2014.06.002 24952334

[B152] Rubin G. M. Spradling A. C. (1982). Genetic transformation of *Drosophila* with transposable element vectors. Science 218, 348–353. 10.1126/science.6289436 6289436

[B153] Saini N. Oelhafen S. Hua H. Georgiev O. Schaffner W. Büeler H. (2010). Extended lifespan of *Drosophila* parkin mutants through sequestration of redox-active metals and enhancement of anti-oxidative pathways. Neurobiol. Dis. 40, 82–92. 10.1016/j.nbd.2010.05.011 20483372

[B154] Saini N. Schaffner W. (2010). Zinc supplement greatly improves the condition of parkin mutant *Drosophila*. Biol. Chem. 391, 513–518. 10.1515/bc.2010.052 20302514

[B155] Sanokawa-Akakura R. Cao W. Allan K. Patel K. Ganesh A. Heiman G.. (2010). Control of Alzheimer's amyloid beta toxicity by the high molecular weight immunophilin FKBP52 and copper homeostasis in *Drosophila*. PLoS ONE 5:e8626. 10.1371/journal.pone.0008626 20084280 PMC2801609

[B156] Sarov M. Barz C. Jambor H. Hein M. Y. Schmied C. Suchold D.. (2016). A genome-wide resource for the analysis of protein localisation in *Drosophila*. Elife 5:e12068. 10.7554/eLife.12068 26896675 PMC4805545

[B157] Schmucker S. Martelli A. Colin F. Page A. Wattenhofer-Donzé M. Reutenauer L.. (2011). Mammalian frataxin: an essential function for cellular viability through an interaction with a preformed ISCU/NFS1/ISD11 iron-sulfur assembly complex. PLoS ONE 6:e16199. 10.1371/journal.pone.0016199 21298097 PMC3027643

[B158] Schofield R. M. Postlethwait J. H. Lefevre H. W. (1997). MeV-ion microprobe analyses of whole *Drosophila* suggest that zinc and copper accumulation is regulated storage not deposit excretion. J. Exp. Biol. 200, 3235–3243. 9364029 10.1242/jeb.200.24.3235

[B159] Sellami A. Wegener C. Veenstra J. A. (2012). Functional significance of the copper transporter ATP7 in peptidergic neurons and endocrine cells in *Drosophila melanogaster*. FEBS Lett. 586, 3633–3638. 10.1016/j.febslet.2012.08.009 22981378

[B160] Selvaraj A. Balamurugan K. Yepiskoposyan H. Zhou H. Egli D. Georgiev O.. (2005). Metal-responsive transcription factor (MTF-1) handles both extremes, copper load and copper starvation, by activating different genes. Genes Dev. 19, 891–896. 10.1101/gad.1301805 15833915 PMC1080128

[B161] Sensi S. L. Paoletti P. Bush A. I. Sekler I. (2009). Zinc in the physiology and pathology of the CNS. Nat. Rev. Neurosci. 10, 780–791. 10.1038/nrn2734 19826435

[B162] Sensi S. L. Paoletti P. Koh J.-Y. Aizenman E. Bush A. I. Hershfinkel M. (2011). The neurophysiology and pathology of brain zinc. J. Neurosci. 31, 16076–16085. 10.1523/JNEUROSCI.3454-11.2011 22072659 PMC3223736

[B163] Singh S. K. Sinha P. Mishra L. Srikrishna S. (2013). Neuroprotective role of a novel copper chelator against Aβ 42 induced neurotoxicity. Int. J. Alzheimers Dis. 2013:567128. 10.1155/2013/567128 24159420 PMC3789492

[B164] Soriano S. Calap-Quintana P. Llorens J. V. Al-Ramahi I. Gutiérrez L. Martínez-Sebastián M. J.. (2016). Metal homeostasis regulators suppress FRDA phenotypes in a *Drosophila* model of the disease. PLoS ONE 11:e0159209. 10.1371/journal.pone.0159209 27433942 PMC4951068

[B165] Soriano S. Llorens J. V. Blanco-Sobero L. Gutiérrez L. Calap-Quintana P. Morales M. P.. (2013). Deferiprone and idebenone rescue frataxin depletion phenotypes in a *Drosophila* model of Friedreich's ataxia. Gene 521, 274–281. 10.1016/j.gene.2013.02.049 23542074

[B166] Southall T. D. Gold K. S. Egger B. Davidson C. M. Caygill E. E. Marshall O. J.. (2013). Cell-type-specific profiling of gene expression and chromatin binding without cell isolation: assaying RNA Pol II occupancy in neural stem cells. Dev. Cell 26, 101–112. 10.1016/j.devcel.2013.05.020 23792147 PMC3714590

[B167] Southon A. Burke R. Camakaris J. (2013a). What can flies tell us about copper homeostasis? Metallomics 5, 1346–1356. 10.1039/c3mt00105a. 23903872

[B168] Southon A. Burke R. Norgate M. Batterham P. Camakaris J. (2004). Copper homoeostasis in *Drosophila melanogaster* S2 cells. Biochem. J. 383, 303–309. 10.1042/BJ20040745 15239669 PMC1134071

[B169] Southon A. Farlow A. Norgate M. Burke R. Camakaris J. (2008). Malvolio is a copper transporter in *Drosophila melanogaster*. J. Exp. Biol. 211, 709–716. 10.1242/jeb.014159 18281333

[B170] Southon A. Greenough M. A. Ganio G. Bush A. I. Burke R. Camakaris J. (2013b). Presenilin promotes dietary copper uptake. PLoS ONE 8:e62811. 10.1371/journal.pone.0062811 23667524 PMC3646984

[B171] Southon A. Greenough M. Hung Y. H. Norgate M. Burke R. Camakaris J. (2011). The ADP-ribosylation factor 1 (Arf1) is involved in regulating copper uptake. Int. J. Biochem. Cell Biol. 43, 146–153. 10.1016/j.biocel.2010.10.012 21034850

[B172] Southon A. Palstra N. Veldhuis N. Gaeth A. Robin C. Burke R.. (2010). Conservation of copper-transporting P(IB)-type ATPase function. Biometals 23, 681–694. 10.1007/s10534-010-9332-2 20372979

[B173] Stathakis D. G. Burton D. Y. McIvor W. E. Krishnakumar S. Wright T. R. O'Donnell J. M. (1999). The catecholamines up (Catsup) protein of *Drosophila melanogaster* functions as a negative regulator of tyrosine hydroxylase activity. Genetics 153, 361–382. 10471719 10.1093/genetics/153.1.361 PMC1460756

[B174] Steiger D. Fetchko M. Vardanyan A. Atanesyan L. Steiner K. Turski M. L.. (2010). The *Drosophila* copper transporter Ctr1C functions in male fertility. J. Biol. Chem. 285, 17089–17097. 10.1074/jbc.M109.090282 20351114 PMC2878003

[B175] Stiller I. Lizák B. Bánhegyi G. (2014). Physiological functions of presenilins; beyond γ-secretase. Curr. Pharm. Biotechnol. 15, 1019–1025. 10.2174/1389201015666141122204139 25420727

[B176] Stuart G. W. Searle P. F. Palmiter R. D. (1985). Identification of multiple metal regulatory elements in mouse metallothionein-I promoter by assaying synthetic sequences. Nature 317, 828–831. 10.1038/317828a0 4058587

[B177] Tejeda-Guzmán C. Rosas-Arellano A. Kroll T. Webb S. M. Barajas-Aceves M. Osorio B. (2017). Zinc storage granules in the Malpighian tubules of *Drosophila melanogaster*. BioRxiv. 10.1101/159558 PMC5897703 29367274

[B178] Ternes A. P. Zemolin A. P. da Cruz L. C. da Silva G. F. Saidelles A. P. F. de Paula M. T.. (2014). *Drosophila melanogaster*-an embryonic model for studying behavioral and biochemical effects of manganese exposure. EXCLI J. 13, 1239–1253. 26417337 PMC4464430

[B179] Terrin G. Berni Canani R. Di Chiara M. Pietravalle A. Aleandri V. Conte F.. (2015). Zinc in early life: a key element in the fetus and preterm neonate. Nutrients 7, 10427–10446. 10.3390/nu7125542 26690476 PMC4690094

[B180] Tsunemi T. Krainc D. (2014). Zn2? dyshomeostasis caused by loss of ATP13A2/PARK9 leads to lysosomal dysfunction and alpha-synuclein accumulation. Hum. Mol. Genet. 23, 2791–2801. 10.1093/hmg/ddt572 24334770 PMC4014186

[B181] Turski M. L. Brady D. C. Kim H. J. Kim B.-E. Nose Y. Counter C. M.. (2012). A novel role for copper in Ras/mitogen-activated protein kinase signaling. Mol. Cell. Biol. 32, 1284–1295. 10.1128/MCB.05722-11 22290441 PMC3302449

[B182] Turski M. L. Thiele D. J. (2007). *Drosophila* Ctr1A functions as a copper transporter essential for development. J. Biol. Chem. 282, 24017–24026. 10.1074/jbc.M703792200 17573340

[B183] Vallee B. L. Falchuk K. H. (1993). The biochemical basis of zinc physiology. Physiol. Rev. 73, 79–118. 8419966 10.1152/physrev.1993.73.1.79

[B184] van Doren M. (2003). fear of intimacy encodes a novel transmembrane protein required for gonad morphogenesis in *Drosophila*. Development 130, 2355–2364. 10.1242/dev.00454 12702650

[B185] Venken K. J. Schulze K. L. Haelterman N. A. Pan H. He Y. Evans-Holm M.. (2011). MiMIC: a highly versatile transposon insertion resource for engineering *Drosophila melanogaster* genes. Nat. Methods 8, 737–743. 10.1038/nmeth.1662 21985007 PMC3191940

[B186] Wang J. Binks T. Warr C. G. Burke R. (2014). Vacuolar-type H^+^-ATPase subunits and the neurogenic protein big brain are required for optimal copper and zinc uptake. Metallomics 6, 2100–2108. 10.1039/C4MT00196F 25209718

[B187] Wang X. Wu Y. Zhou B. (2009). Dietary zinc absorption is mediated by ZnT1 in *Drosophila melanogaster*. FASEB J. 23, 2650–2661. 10.1096/fj.08-126649 19325039

[B188] Wang Y. Wimmer U. Lichtlen P. Inderbitzin D. Stieger B. Meier P. J.. (2004). Metal-responsive transcription factor-1 (MTF-1) is essential for embryonic liver development and heavy metal detoxification in the adult liver. FASEB J. 18, 1071–1079. 10.1096/fj.03-1282com 15226267

[B189] Wang Z. Ferdousy F. Lawal H. Huang Z. Daigle J. G. Izevbaye I.. (2011). Catecholamines up integrates dopamine synthesis and synaptic trafficking. J. Neurochem. 119, 1294–1305. 10.1111/j.1471-4159.2011.07517.x 21985068 PMC3233821

[B190] White A. R. Multhaup G. Maher F. Bellingham S. Camakaris J. Zheng H.. (1999). The Alzheimer's disease amyloid precursor protein modulates copper-induced toxicity and oxidative stress in primary neuronal cultures. J. Neurosci. 19, 9170–9179. 10531420 10.1523/JNEUROSCI.19-21-09170.1999 PMC6782934

[B191] Whitnall M. Suryo Rahmanto Y. Sutak R. Xu X. Becker E. M. Mikhael M. R.. (2008). The MCK mouse heart model of Friedreich's ataxia: alterations in iron-regulated proteins and cardiac hypertrophy are limited by iron chelation. Proc. Natl. Acad. Sci. U.S.A. 105, 9757–9762. 10.1073/pnas.0804261105 18621680 PMC2474513

[B192] Wieschaus E. Nüsslein-Volhard C. Jürgens G. (1984). Mutations affecting the pattern of the larval cuticle in *Drosophila melanogaster*: III. Zygotic loci on the X-chromosome and fourth chromosome. Wilhelm Roux Arch. Dev. Biol. 193, 296–307. 10.1007/BF00848158 28305339

[B193] Wright T. R. F. (1987). The Genetics Of Biogenic Amine Metabolism, Sclerotization, And Melanization In *Drosophila Melanogaster*^**^This review is dedicated to Professor Ernst Caspari in recognition of his pioneering research in biochemical genetics, in Molecular Genetics of Development, ed Scandalios J. G. (San Diego, CA: Academic Press), 127–222. 3124532

[B194] Wu Z. Du Y. Xue H. Wu Y. Zhou B. (2012). Aluminum induces neurodegeneration and its toxicity arises from increased iron accumulation and reactive oxygen species (ROS) production. Neurobiol. Aging 33, 199, e1–12. 10.1016/j.neurobiolaging.2010.06.018 20674094

[B195] Xiao G. Fan Q. Wang X. Zhou B. (2013). Huntington disease arises from a combinatory toxicity of polyglutamine and copper binding. Proc. Natl. Acad. Sci. U.S.A. 110, 14995–15000. 10.1073/pnas.1308535110 23980182 PMC3773747

[B196] Xiao G. Wan Z. Fan Q. Tang X. Zhou B. (2014). The metal transporter ZIP13 supplies iron into the secretory pathway in *Drosophila melanogaster*. Elife 3:e03191. 10.7554/eLife.03191 25006035 PMC4130162

[B197] Xie J. Owen T. Xia K. Singh A. V. Tou E. Li L.. (2015). Zinc inhibits Hedgehog autoprocessing: linking zinc deficiency with Hedgehog activation. J. Biol. Chem. 290, 11591–11600. 10.1074/jbc.M114.623264 25787080 PMC4416862

[B198] Xu Z. Tito A. J. Rui Y.-N. Zhang S. (2015). Studying polyglutamine diseases in *Drosophila*. Exp. Neurol. 274, 25–41. 10.1016/j.expneurol.2015.08.002 26257024 PMC4644473

[B199] Yepiskoposyan H. Egli D. Fergestad T. Selvaraj A. Treiber C. Multhaup G.. (2006). Transcriptome response to heavy metal stress in *Drosophila* reveals a new zinc transporter that confers resistance to zinc. Nucleic Acids Res. 34, 4866–4877. 10.1093/nar/gkl606 16973896 PMC1635269

[B200] Yin S. Qin Q. Zhou B. (2017). Functional studies of *Drosophila* zinc transporters reveal the mechanism for zinc excretion in Malpighian tubules. BMC Biol. 15:12. 10.1186/s12915-017-0355-9 28196538 PMC5309981

[B201] Zalewski P. D. Forbes I. J. Betts W. H. (1993). Correlation of apoptosis with change in intracellular labile Zn(II) using zinquin (2-methyl-8-p-toluenesulphonamido-6-quinolyloxy)acetic acid, a new specific fluorescent probe for Zn(II). Biochem. J. 296(Pt 2), 403–408. 10.1042/bj2960403 8257431 PMC1137710

[B202] Zhang B. Egli D. Georgiev O. Schaffner W. (2001). The *Drosophila* homolog of mammalian zinc finger factor MTF-1 activates transcription in response to heavy metals. Mol. Cell. Biol. 21, 4505–4514. 10.1128/MCB.21.14.4505-4514.2001 11416130 PMC87110

[B203] Zhang X. Koolhaas W. H. Schnorrer F. (2014). A versatile two-step CRISPR- and RMCE-based strategy for efficient genome engineering in *Drosophila*. G3 (Bethesda) 4, 2409–2418. 10.1534/g3.114.013979 25324299 PMC4267936

[B204] Zhou H. Cadigan K. M. Thiele D. J. (2003). A copper-regulated transporter required for copper acquisition, pigmentation, and specific stages of development in *Drosophila melanogaster*. J. Biol. Chem. 278, 48210–48218. 10.1074/jbc.M309820200 12966081

[B205] Zhou S. Luoma S. E. St Armour G. E. Thakkar E. Mackay T. F. C. Anholt R. R. H. (2017). A *Drosophila* model for toxicogenomics: genetic variation in susceptibility to heavy metal exposure. PLoS Genet. 13:e1006907. 10.1371/journal.pgen.1006907 28732062 PMC5544243

[B206] Zhu Z.-J. Wu K.-C. Yung W.-H. Qian Z.-M. Ke Y. (2016). Differential interaction between iron and mutant alpha-synuclein causes distinctive Parkinsonian phenotypes in *Drosophila*. Biochim. Biophys. Acta 1862, 518–525. 10.1016/j.bbadis.2016.01.002 26769358

[B207] Zlatic S. Comstra H. S. Gokhale A. Petris M. J. Faundez V. (2015). Molecular basis of neurodegeneration and neurodevelopmental defects in Menkes disease. Neurobiol. Dis. 81, 154–161. 10.1016/j.nbd.2014.12.024 25583185 PMC4499018

